# Anion-adaptive crystalline cationic material for ^99^TcO_4_^−^ trapping

**DOI:** 10.1038/s41467-019-09504-3

**Published:** 2019-04-04

**Authors:** Lei Mei, Fei-ze Li, Jian-hui Lan, Cong-zhi Wang, Chao Xu, Hao Deng, Qun-yan Wu, Kong-qiu Hu, Lin Wang, Zhi-fang Chai, Jing Chen, John K. Gibson, Wei-qun Shi

**Affiliations:** 1 Laboratory of Nuclear Energy Chemistry, Institute of High Energy Physics, Chinese Academy of Sciences, Beijing, 100049 China; 2 Nuclear Chemistry and Chemical Engineering Division, Institute of Nuclear and New Energy Technology, Tsinghua University, Beijing, 100084 China; 3 Engineering Laboratory of Nuclear Energy Materials, Ningbo Institute of Industrial Technology, Chinese Academy of Sciences, Ningbo, Zhejiang 315201 China; 4 Chemical Sciences Division, Lawrence Berkeley National Laboratory, Berkeley, California 94720 USA

## Abstract

Efficient anion recognition is of great significance for radioactive ^99^TcO_4_^−^ decontamination, but it remains a challenge for traditional sorbents. Herein, we put forward a tactic using soft crystalline cationic material with anion-adaptive dynamics for ^99^TcO_4_^−^ sequestration. A cucurbit[8]uril-based supramolecular metal-organic material is produced through a multi-component assembly strategy and used as a sorbent for effective trapping of TcO_4_^−^. Excellent separation of TcO_4_^−^/ReO_4_^−^ is demonstrated by fast removal kinetics, good sorption capacity and high distribution coefficient. Remarkably, the most superior selectivity among metal-organic materials reported so far, together with good hydrolytic stability, indicates potential for efficient TcO_4_^−^ removal. The structure incorporating ReO_4_^−^ reveals that the supramolecular framework undergoes adaptive reconstruction facilitating the effective accommodation of TcO_4_^−^/ReO_4_^−^. The results highlight opportunities for development of soft anion-adaptive sorbents for highly selective anion decontamination.

## Abstract (web summary)

Efficient anion recognition and trapping is of great significance for anion-specific separation processes but the design of an anion-adaptive sorbents remains a challenge. Here the authors use a cucurbit[8]uril based soft cationic supramolecular material as efficient sorbent for TcO_4_^−^ ions.

## Introduction

^99^Tc, a long-lived (*t*_1/2_ = 2.13 × 10^5^ y) radioisotope of technetium, is an abundant and problematic nuclear waste component and potent radioactive pollution source^1,2^. Complex chemical behavior of ^99^Tc hampers separation of uranium and plutonium during reprocessing of spent nuclear fuel, and high volatility of ^99^Tc species (Tc_2_O_7_) constrains incorporation into glass waste forms via high-temperature vitrification. ^99^Tc, as a stable TcO_4_^−^ in its dominant + 7 oxidation state, is highly water soluble and can migrate readily in the environment, thereby posing severe environmental risks. Therefore, efficient capture of radioactive ^99^Tc has received considerable attention for both nuclear waste management and contaminant remediation purposes.

Solvent extraction and ion exchange are two well-established effective methods for removal of TcO_4_^−^ from aqueous media^3–6^. In solvent extraction, extractants with anion recognition capability can achieve high selectivity for TcO_4_^− ^^4,7–9^, but practical applications are limited by cost and inefficiency. Ion exchange is developed as an alternative of traditional extraction. Despite the ease of implementation and the expected efficient recovery of TcO_4_^−^ based on ion-exchange method^5,10^, the total performance of sorbent materials used seems not to be competent. For example, most traditional polymeric materials exhibit slow anion exchange kinetics and poor radiation resistance^11,12^, while inorganic cationic materials such as layered double hydroxide (LDH)^13^, sulfides^14^, and borates^15,16^ exhibit low sorption capacity and poor selectivity. The emergence of hydrolytically stable cationic metal–organic materials (MOMs) has led to potent applications for capture of oxyanion pollutants^3,17–20^. High porosity, structural diversity, and functional tunability^21–23^ render these hybrid materials as promising candidates for TcO_4_^−^ removal^24,25^. However, there is still demand for improvement in terms of selectivity of TcO_4_^−^ sorbents, with enhanced discrimination for TcO_4_^−^/ReO_4_^−^ over other anions as a particularly desirable attribute.

**Fig. 1:**
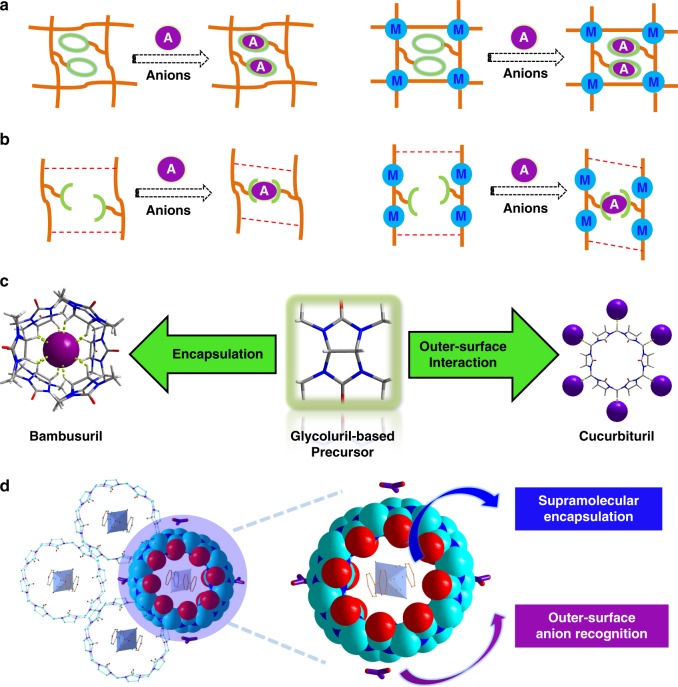
Design strategies for introducing anion-adaptive capability to sorbent materials. **a** Direct modification of organic polymers or metal–organic sorbents with macrocyclic motifs bearing anion-recognition ability (orange lines: backbones of solid sorbents; green circles: anion receptors; purple balls marked as “A”: anions; blue balls marked as “M”: metal nodes). **b** Supramolecular assembly of smart polymers or metal–organic sorbents exhibiting anion-adaptive behaviors (orange lines: backbones of solid sorbents; green curves: anion-recognition sites; purple circles: anions; blue balls: metal nodes). **c** Glycoluril-based macrocyclic hosts for anion recognition through two different modes (purple balls: anions). **d** Dual roles of CB8 in accomplishing both the construction of a supramolecular network by encapsulation and providing outer-surface interaction sites for anion recognition: left, a full view of CB8-based supramolecular framework; right: enlarged diagram of CB8 showing its interactions with the surrounding components (red balls: O atoms; light-blue balls: C atom; dark blue balls: N atoms)

There is no doubt that anion receptors^7,9,26–29^ of TcO_4_^−^ have the best ion selectivity. A rational approach for improving the selectivity of solid sorbents is to give sorbent materials an ideal ion-recognition capability by direct functionalization of traditional solid sorbents with covalently attached anion receptors (Fig. 1a)^30,31^, but unfortunately, chemical modification method generally suffers from the necessity for elaborate syntheses and possible deactivation of functional recognition groups after implanting in bulk materials. Herein, inspired by molecular recognition of anion receptors, we put forward an alternative tactic that circumvents this drawback via an easily prepared anion-adaptive sorbent material that can behave like an anion receptor itself (Fig. 1b)^32–36^. Conceptually, this soft sorbent material is capable of dynamically tuning the structural arrangement of its framework in response to different anions, enabling attainment of an optimized pore size and shape match for maximum interactions with, and the resulting selectivity for target anions such as TcO_4_^−^. Specifically, the desirable anion-adaptive sorbent material should have two crucial attributes of structural dynamics and anion-responsive capability, which can be easily achieved in self-organization-based supramolecular materials bearing recognition sites^37,38^. Glycoluril derivatives containing abundant –CH or –CH_2_ motifs as potent anion-recognition sites, among which *endo*-type bambusurils can serve as anion receptors^39–41^, while *exo*-type cucurbiturils exhibit anion-binding affinity through outer-surface interaction (Fig. 1c), can be used to prepare a class of anion-adaptive cationic materials for specific anion removal^42–45^. A multicomponent assembly strategy is proposed for synthesizing such sorbents based on a versatile glycoluril-based macrocyclic host, cucurbit[8]uril (CB8). The CB8 macrocycle used here plays a vital role in accomplishing both the construction of a supramolecular network^46–48^ and anion recognition (Fig. 1d), and endows both important attributes mentioned above: (a) abundant CH and CH_2_ groups on its waist for outer-surface hydrogen-bonding recognition to promote the TcO_4_^−^ capture; (b) flexibility of the CB8 encapsulation motif allowing dynamics of the overall supramolecular framework^49–51^.

In this work, a CB8-based cationic supramolecular metal–organic framework, SCP-IHEP-1 ([Cu((bpy)_2_@CB8)(H_2_O)_4_](NO_3_)_2_·18H_2_O), is constructed by the supramolecular collaborative assembly. As expected, this material is demonstrated to be an efficient and selective sorbent capable of reversibly sequestrating TcO_4_^−^/ReO_4_^−^ by trapping them in specific tetrahedral pores created by CB8 moieties arranged in order. The anion-adaptive capability of this supramolecular sorbent toward effective TcO_4_^−^ recognition resembles the dynamic behavior of the receptor during ion recognition, and can be taken as a representative TcO_4_^−^-specific smart sorbent material.

## Results

### Assembly of SCP-IHEP-1 based on CB8

**Fig. 2:**
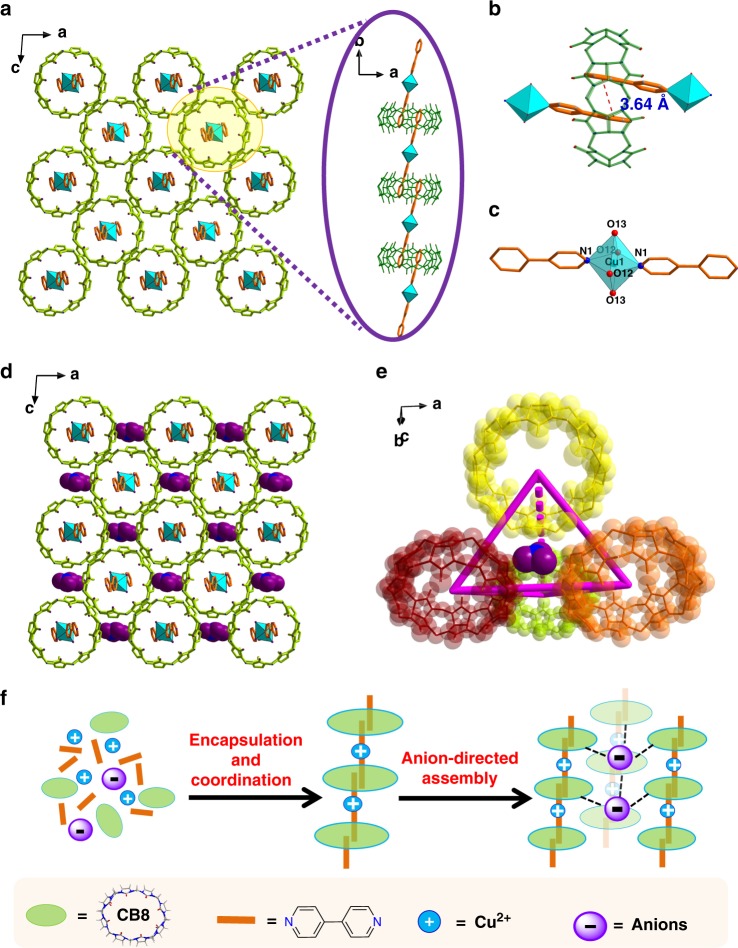
Crystal structure of SCP-IHEP-1. **a** The cationic supramolecular framework (insert: building blocks of the 1D metal–organic polyrotaxane chain, green macrocycles: cucurbit[8]uril (CB8) molecules; skeletons in orange: 4,4′-bipyridine (bpy); polyhedra in mint green: coordination spheres of copper ion; **b** the supramolecular assembly motif of 2bpy@CB8; **c** [Cu(bpy)_2_(H_2_O)_4_]^2+^ coordination motif; **d** nitrate-incorporated supramolecular framework of SCP-IHEP-1 (purple: O atom; blue: N atom); **e** NO_3_^−^ in a tetrahedral coordination cavity; **f** proposed stepwise self-assembly process of SCP-IHEP-1 involving four different components

The cationic supramolecular framework material, SCP-IHEP-1, was synthesized via assembly of CB8, 4,4′-bipyridine (bpy) and Cu(NO_3_)_2_ under hydrothermal conditions. It crystallizes in monoclinic space group *P*2_1_/n (Supplementary Table 1) as pale blue block crystals (Supplementary Figure 1). Crystal structure of SCP-IHEP-1 reveals that all the four components (CB8, bpy, Cu^2+^, and NO_3_^−^) (Supplementary Figure 2) are included during the self-assembly process, and the main building unit of the supramolecular framework of SCP-IHEP-1 (Fig. 2a) is a one-dimensional (1D) metal–organic polyrotaxane chain (Fig. 2b) based on encapsulation motif, 2bpy@CB8, linked by Cu^2+^ (Fig. 1c). Given the potential for competition between metal coordination and supramolecular encapsulation of bpy, one-pot synthesis of SCP-IHEP-1 having both types of connectivities for bpy suggests stepwise assembly, with initial supramolecular encapsulation of bpy in CB8 followed by assembly into a 1D chain via bpy-Cu^2+^ coordination (Supplementary Figure 3). This assembly mechanism was corroborated by an alternative two-step method in which isolation of the dimeric bpy units encapsulated in CB8, 2bpy@CB8, in the form of [(bpy)_2_@CB8]_0.5_·[(bpy)_2_@CB8]_0.5_·19H_2_O (Supplementary Figure 4 and 5) is followed by assembly with the metal ions provided as Cu(NO_3_)_2_. It is notable that, whereas 2G@H encapsulation, where G denotes a guest molecule, and H a host, is common for host CB8^46–48,52^, this motif is rare for neutral guest such as bpy in CB8^53^. Actually, formation of 2bpy@CB8 in aqueous solution involves a large favorable enthalpy change as a result of release of high-energy water (Supplementary Figure 6 and Supplementary Table 2), which should be taken as the main driving force for this encapsulation^54,55^ and results in different degrees of pi–pi stacking (Supplementary Figure 4) and a variety of hydrogen bonds between CB8 and bpy (Supplementary Figure 7 and Supplementary Table 3).

Cationic 1D polyrotaxane chains in SCP-IHEP-1 can further assemble into a three-dimensional (3D) supramolecular framework via cross-linkage of a large number of interchain hydrogen bonds (Supplementary Figure 8 and Supplementary Table 4). The nitrate counterions are located in tetrahedral cavities formed by four neighboring CB8 from the 1D chains (Fig. 2d, e and Supplementary Figure 9), and also contributed a lot to the formation of final supramolecular framework via anion-directed assembly (Fig. 2f). Analysis of the local nitrate anion environment reveals its interaction with only two CB8 macrocycles of the tetrahedral cavity through a limited number of hydrogen bonds (Supplementary Figure 10), suggesting weak interaction with main 1D backbones of SCP-IHEP-1 and the potential for exchange with oxyanions having more favorable interactions, such as TcO_4_^−^/ReO_4_^−^.

In contrast, coordination assembly simply from bpy and Cu(NO_3_)_2_ in the absence of CB8 macrocycles results in a twofold interpenetrating 3D framework based on six-coordinated copper nodes with deformed octahedral geometry (namely as Cu-bpy, see Supplementary Table 1 and Supplementary Figure 11). In addition to the nitrate directly binding to a copper center, there should be a disordered nitrate anion in the pore of 3D framework to balance the charge.

**Fig. 3:**
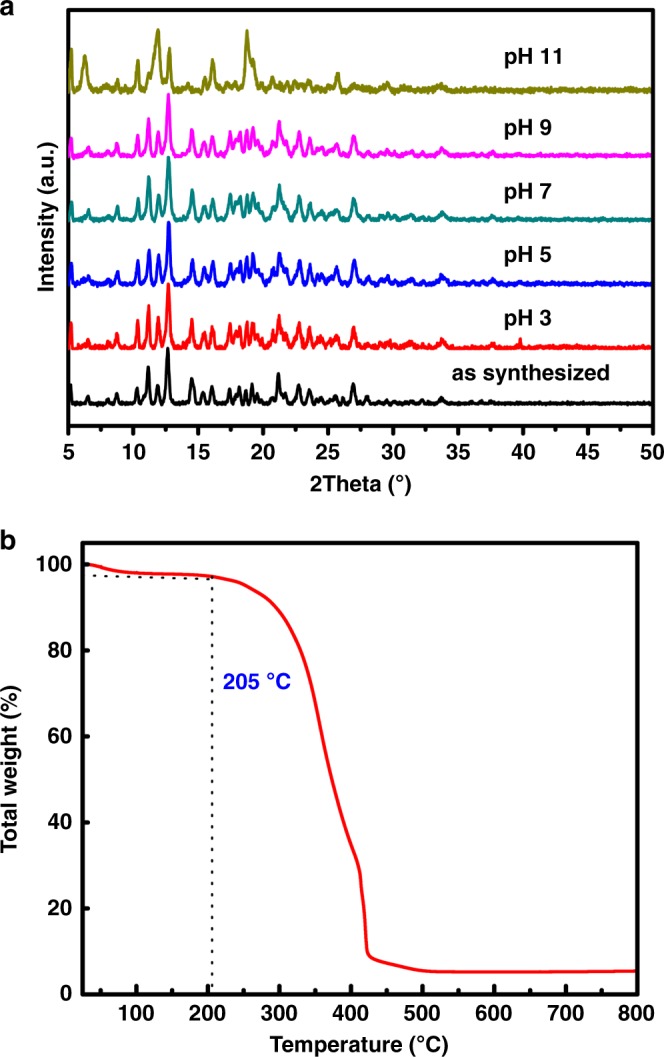
Hydrolytic stability and thermal stability of SCP-IHEP-1. **a** PXRD patterns of SCP-IHEP-1 after soaking in aqueous solutions with varying pH values ranging from 3 to 11 for 12 hours at 298 K. **b** Thermogravimetric analysis (TGA) in air for SCP-IHEP-1

After exposure of SCP-IHEP-1 crystals for 12 h at 298 K to aqueous solutions with pH values ranging from 3 to 11, the structure as determined by powder X-ray diffraction (PXRD) remained essentially unchanged (Fig. 3a). The results suggest thermal and hydrolytic stability, despite the flexible supramolecular framework. Meanwhile, in contrast to significant dehydration of Cu-bpy at low temperatures (Supplementary Figure 12), SCP-IHEP-1 did not undergo any significant decomposition until over 205 °C (Fig. 3b), suggesting its high thermal stability.

### Sorption performance for ReO_4_^−^ removal

**Fig. 4:**
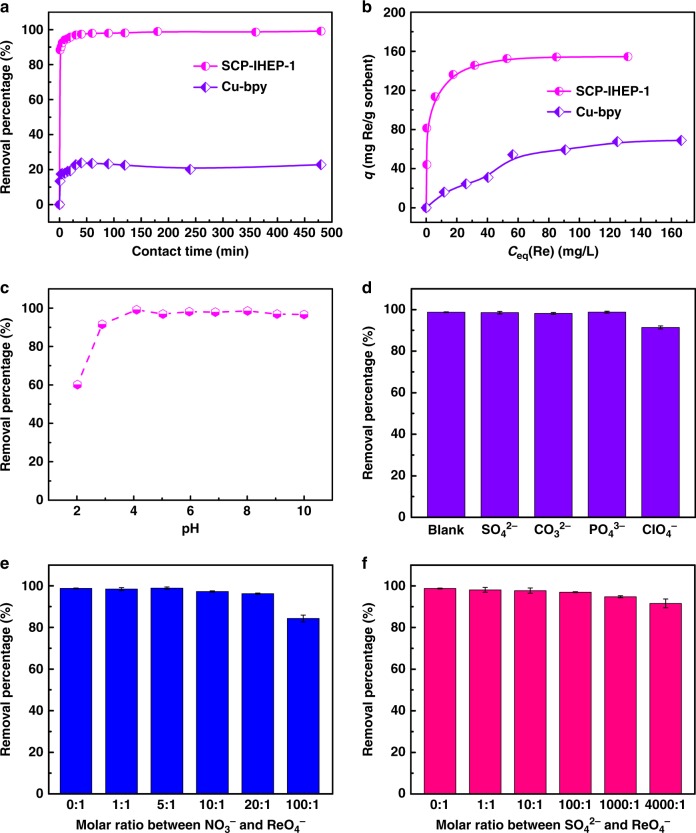
Performance of SCP-IHEP-1 for ReO_4_^−^ removal. **a** Sorption kinetics with that of Cu-bpy used as a comparison; **b** sorption isotherms with that of Cu-bpy used as a comparison; **c** effect of pH on the removal percentage of ReO_4_^−^ (*c*_0_ = 44.6 mg L^−1^, *T* = 300 K, *t* = 12 h, *V* = 8 mL, and *m* = 4 mg); **d** effect of competing anions on the ReO_4_^−^ exchange (*c*_0_ = 45.0 mg L^−1^, *t* = 12 h, *T* = 300 K, *V* = 8 mL, *m* = 4 mg, and pH = 6.92); **e** effect of excess NO_3_^−^ on the ReO_4_^−^ exchange (*c*_0_ = 45.0 mg L^−1^, *t* = 12 h, *T* = 300 K, *V* = 8 mL, *m* = 4 mg, and pH = 6.85); **f** effect of excess SO_4_^2−^ on the ReO_4_^−^ exchange (*c*_0_ = 45.0 mg L^−1^, *t* = 12 h, *T* = 300 K, *V* = 8 mL, *m* = 4 mg, and pH = 7.05). Error bars represent the s.d. of uncertainty for each point

ReO_4_^−^ was initially used as a nonradioactive structural analog of ^99^TcO_4_^−^ to assess anion exchange of SCP-IHEP-1. Batch kinetics experiment shows that removal of ReO_4_^−^ by SCP-IHEP-1 follows the pseudo-first-order model (Supplementary Figure 13 and Supplementary Table 5), and is achieved to 88% removal at 1 min and to over 95% after 10 min (Fig. 4a). The fast kinetics of ReO_4_^−^ exchange by SCP-IHEP-1 is superior to those of other cationic metal–organic materials such as SLUG-21^20^, UiO-66-NH_3_^+^^17^, and Ni(II)-based MOF^19^, all of which take over 24 h to reach exchange equilibrium for sequestering ReO_4_^−^. It is notably that, SCP-IHEP-1 also shows faster removal rate and much higher removal ratio than its CB8-free counterpart Cu-bpy (an equilibrium time of 30 min and a final removal ratio of 20%, Supplementary Figure 14 and Supplementary Table 6).

As revealed by the sorption isotherm experiment (Fig. 4b), the calculated maximum sorption capacity of SCP-IHEP-1 based on the Langmuir model is 157 mg Re g^−1^ sorbent corresponding to 211 mg ReO_4_^−^ g^−1^ sorbent (Supplementary Figure 15 and Supplementary Table 7), which is higher than those for LDH (130 mg ReO_4_^−^ g^−1^)^13^, NDTB-1 (49 mg ReO_4_^−^ g^−1^)^15,]^^16^, and UiO-66-NH_3_^+^ (159 mg ReO_4_^−^ g^−1^)^17^. Assuming that all the nitrate ions can be exchanged, the sorption capacity of SCP-IHEP-1 for ReO_4_^−^ observed here reaches to as high as 93% of the theoretical value (226 mg g^−1^), suggesting its nearly perfect exchange tendency for ReO_4_^−^. Moreover, the distribution coefficient (*K*_d_) of SCP-IHEP-1 toward ReO_4_^−^ is 2.6 × 10^5^ mL g^−1^ (Supplementary Table 8), which is also comparable to recently emerging high-performance cationic MOFs, SCU-100, and SCU-101 (Table 1)^24,25^, and ensures the decontamination depth of ReO_4_^−^. In contrast, ReO_4_^−^ removal by CB8-free Cu-bpy can only achieve a poor separation efficiency (*K*_d_: ~1 × 10^3^ mL g^−1^, Supplementary Table 8), which is two orders of magnitude lower than that of SCP-IHEP-1. Correspondingly, the derived maximum sorption capacity of 138 mg ReO_4_^−^ g^−1^ (Supplementary Figure 16 and Supplementary Table 9) only reaches 14% of the theoretical value (950 mg ReO_4_^−^ g^−1^ assuming all the anions could be exchangeable.

**Table 1:** Comparison of ReO_4_^−^ removal performance between different cationic materials

Materials	*K*_d_ (mL g^−1^)	Removal rate (ReO_4_^−^-NO_3_^−^) (%)	Removal rate (ReO_4_^−^-SO_4_^2−^) (%)	Removal rate (ReO_4_^−^-PO_4_^3−^) (%)	Removal rate (ReO_4_^−^-ClO_4_^−^) (%)	Ref.
LDH	262	–	–	–	–	^25^
NDTB-1	652	–	–	–	–	^25^
PAF-1-F	2.55 × 10^5^		19	21	–	^56^
UiO-66-NH_3_^+^	–	–	50	15	21	^17^
SCU-100	3.3 × 10^5^	–	98.5	98.7	–	^25^
SCU-101	7.5 × 10^5^	91.7	85.6	89.2	87.2	^24^
SCP-IHEP-1	2.6 × 10^5^	98.4	98.5	98.8	91.4	This work

In terms of structural reversibility, over 96% of sorbed ReO_4_^−^ could be exchanged with NO_3_^−^ in a desorption solution of 0.5 M NaNO_3_, and the regenerated material retains over 92% of removal percentage after two cycles. The removal percentage of SCP-IHEP-1 for ReO_4_^−^ keeps at a high level (>96%) within a wide pH range of 4−10 (Fig. 4c) and remains up to 60% at more acidic media with a pH value of 2, revealing a good hydrolytic stability and high separation capability. Studies on the effect of temperature (Supplementary Figure 17) indicates that the present anion exchange may be an exothermic process with an enthalpy change of −25.76 kJ mol^−1^ (Supplementary Table 10).

### Uptake selectivity

Studies on ReO_4_^−^ exchange selectivity show that SCP-IHEP-1 still selectively removes ReO_4_^−^ in the presence of one equivalent of several competing anions including NO_3_^−^, SO_4_^2−^, CO_3_^2−^, PO_4_^3−^, or ClO_4_^−^ (Fig. 4d). Although ReO_4_^−^ removal diminishes somewhat when competing with structurally similar ClO_4_^−^, it remains as high as 91% (Supplementary Table 11). Under similar conditions, ReO_4_^−^ removal by SCU-101^24^ does not exceed 90%, and removal by PAF-1-F^56^ is only ca. 20% in the presence of SO_4_^2−^ or PO_4_^3−^.

Given the high concentration of nitrate ion in high level nuclear waste stream, the competing effect of excess nitrate anions is critical during TcO_4_^−^ removal. Moreover, for certain types of nuclear waste, high-concentration SO_4_^2−^ is also another potent competing anions. Therefore, ReO_4_^−^ removal with higher concentrations of competing NO_3_^−^ and SO_4_^2−^ were further studied to check the uptake selectivity of ReO_4_^−^. As shown in Fig. 3e, removal of ReO_4_^−^ remains as high as 96% for a molar ratio of NO_3_^−^ to ReO_4_^−^ of 20:1, and is 84% for a ratio of 100:1 (Supplementary Table 12). Remarkably, an increase in SO_4_^2−^ has little effect on the uptake of ReO_4_^−^ (Fig. 3f), with removal falling only from 98 to 92% when the SO_4_^2−^:ReO_4_^−^ ratio increases from 1:1 to 4000:1 (Supplementary Table 13).

Removal selectivity of SCP-IHEP-1 toward TcO_4_^−^/ReO_4_^−^ can be partially ascribed to its inherent feature of inorganic–organic hybrid material based on multi-component collaborative assembly. Generally, anions with higher charge density such as SO_4_^2−^ often have better uptake than those with lower charge density (TcO_4_^−^/ReO_4_^−^) during the sorption process with inorganic anion sorbents^13,15,16^. However, this order always is reversed for organic polymers and inorganic–organic hybrid materials, which is taken as a Hofmeister phenomenon^57^. This Hofmeister behavior might be originated from the hydrophobic nature of organic backbones of these materials, as evidenced by the methylene/methylidyne-rich tetrahedral pores of SCP-IHEP-1.^3^ A similar trend is observed for other MOMs bearing local hydrophobic cavities or pores^24,25,58^. That is to say, considering the differences in hydration energy of anions, the preference for larger poorly hydrated ReO_4_^−^ anions over NO_3_^−^ or SO_4_^2−^ reflects the important role of hydration/dehydration in the anion exchange, which is consistent with the exothermic feature of exchange observed above.

### TcO_4_^−^ removal from simulated nuclear wastes

**Fig. 5:**
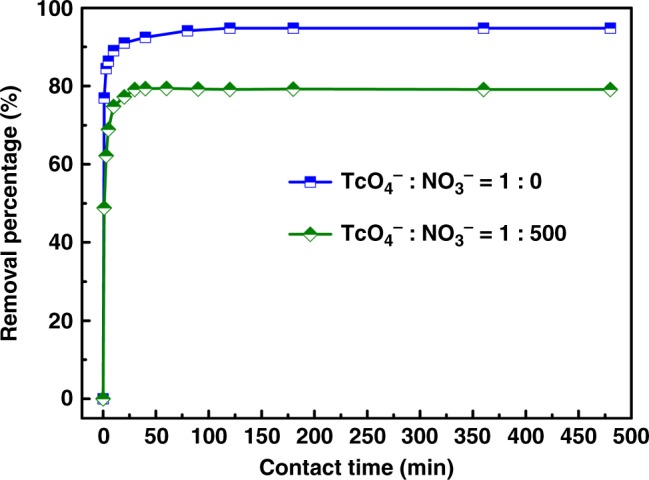
Removal of TcO_4_^−^ as a function of contact time in the absence of competing anion NO_3_^−^ (blue line; TcO_4_^−^: NO_3_^−^ = 1: 0, *c*_0_ = 10 mg L^−1^, *V* = 40 mL, *m* = 20 mg, and pH = 7.00) and in the presence of 0.03 M NO_3_^−^ (green line; TcO_4_^−^: NO_3_^−^ = 1: 500, *c*_0_ = 10 mg L^−1^, *V* = 10 mL, *m* = 5 mg, and pH = 6.89). Error bars represent the s.d. of uncertainty for each point

The overall selectivity of SCP-IHEP-1 toward ReO_4_^−^ against NO_3_^−^ and SO_4_^2−^ observed here is better than those of MOF-typed (SCU-101)^25^ and polymeric network-typed (SCU-CPN-1)^57^ anionic exchange materials with excellent ReO_4_^−^/TcO_4_^−^ removal performance emerging recently, making it a promising candidate for selective sequestration of TcO_4_^−^ from waste solutions, even in the presence of high concentration of competing anions. To assess the potential application of SCP-IHEP-1 in real nuclear solutions containing radioactive TcO_4_^−^, removal experiments for TcO_4_^−^ were also tested. Uptake kinetics of TcO_4_^−^ by SCP-IHEP-1 is as fast as that of ReO_4_^−^, achieving ~80% removal at 1 min and over 90% after 10 min (Fig. 5), and nearly quantitative removal after 2 h. In a simplified simulated waste stream containing 9.8 ppm ^99^TcO_4_^−^ in 0.03 M HNO_3_ (i.e., a NO_3_^−^ concentration ~ 500 times higher than TcO_4_^−^), although the TcO_4_^−^ removal is affected by the high-concentration competing NO_3_^−^, the removal percentage of TcO_4_^−^ is still up to 79.2% using a solid-to-liquid ratio (SLR) of 0.5, which is superior to the removal by SCU-100 (59.3% with a SLR of 1.0)^25^ and SCU-101 (75.2% with a higher SLR of 10)^24^ and represents the best removal performance for ^99^TcO_4_^−^ among cationic MOMs reported so far.

### Mechanism for selective ReO_4_^−^ uptake

**Fig. 6:**
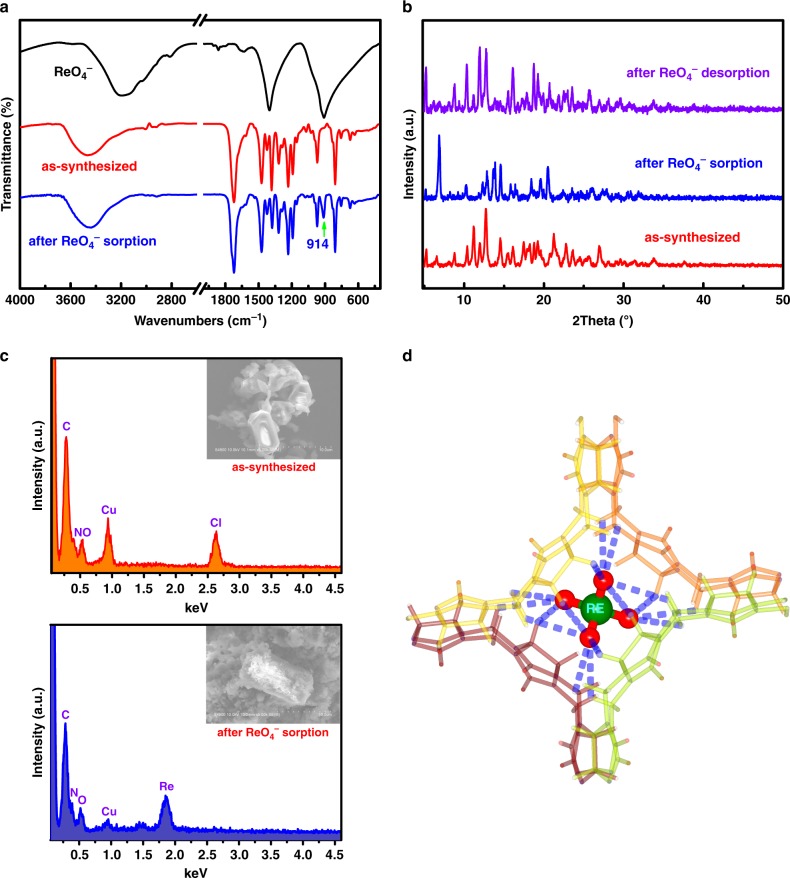
Characterization of SCP-IHEP-1 after ReO_4_^−^ sorption. **a** IR spectra of SCP-IHEP-1 before (red) and after (blue) ReO_4_^−^ exchange; **b** PXRD patterns before ReO_4_^−^ sorption (red), after ReO_4_^−^ sorption (blue) and after material recovery by ReO_4_^−^ desorption in a solution of 0.5 M NaNO_3_ (purple); **c** EDS of SCP-IHEP-1 before (top) and after (bottom) ReO_4_^−^ exchange; **d** ReO_4_^−^ instead of NO_3_^−^ trapped in a tetrahedral coordination cavity after ReO_4_^−^ sorption (four adjacent CB8 molecules are shown in different colors and hydrogen atoms in black)

ReO_4_^−^ (and by inference TcO_4_^−^) exchange of SCP-IHEP-1 was monitored by Fourier transform infrared spectroscopy (FTIR) (Fig. 6a), PXRD patterns (Fig. 6b), and energy dispersive X-ray spectroscopy (EDS) (Fig. 6c). Single crystals of ReO_4_^−^ incorporated material (SCP-IHEP-1-Re) were also obtained and subject to X-ray diffraction structural determination on the Beijing Synchrotron Radiation Facility (BSRF) (Supplementary Figure 18). A comparison of simulated PXRD pattern of crystalline SCP-IHEP-1-Re and actual PXRD of SCP-IHEP-1 after ReO_4_^−^ exchange (Supplementary Figure 19) confirms that they are totally identical to each other, and crystallographic analysis at the molecular level will be very helpful to understand the recognition mechanism of SCP-IHEP-1 toward ReO_4_^−^ as well as TcO_4_^−^. Similarly, ReO_4_^−^ uptake of Cu-bpy was also evidenced by the signals of ReO_4_^−^ or Re in FTIR (Supplementary Figure 20) and EDS (Supplementary Figure 21) after exchange experiments. PXRD comparison (Supplementary Figure 22) between Cu-bpy after ReO_4_^−^ exchange and ReO_4_^−^ incorporated crystalline analog of Cu-bpy (Supplementary Figure 23) suggests a possible ReO_4_^−^-induced transformation of Cu-bpy to Cu-bpy-Re along with significant change of Cu^2+^ coordination sphere and total topological structure, although there might be also a small amount of other undefined products.

**Fig. 7:**
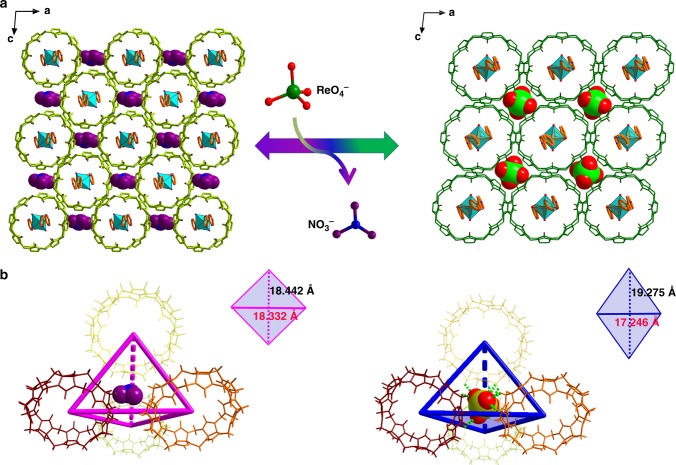
ReO_4_^−^ exchange-triggered structural transformation of SCP-IHEP-1. **a** Single-crystal-to-single-crystal (SCSC) transformation from SCP-IHEP-1 to SCP-IHEP-1-Re upon ReO_4_^−^ incorporation. **b** Adaptive structural transformation of CB8-based tetrahedral cavity after encapsulation of ReO_4_^−^

Analysis on the crystal structure of SCP-IHEP-1-Re reveals that ReO_4_^−^ is trapped in tetrahedral pores surrounded by four adjacent CB8 molecules (Fig. 6d) and fixed by a mass of hydrogen bonds between anion oxygen atoms and outer-surface CH and CH_2_ groups (a total of 15 such bonds with an average O·H distance of 2.71 Å) (Supplementary Table 3). The crystal structure of SCP-IHEP-1-Re reveals close-packing mode similar to SCP-IHEP-1 but with a change of stacking orientation originated from its single-crystal-to-single-crystal (SCSC) transformation upon ReO_4_^−^ incorporation (Fig. 7a). Compared to the coordination environment of NO_3_^−^ in SCP-IHEP-1, encapsulation of ReO_4_^−^ is achieved by the rearrangement of surrounding CB8 moieties with a resulting slight deformation of the tetrahedral pores (Fig. 7b). The above results indicate that the removal of TcO_4_^−^/ReO_4_^−^ by SCP-IHEP-1 is mainly attributed to anion-adaptive reorganization of the CB8-based pores, which can dynamically adapt to the encapsulated anionic guest.

A comparison of this CB8-based MOM sorbent material with Cu-bpy or other CB8-free MOM sorbents material such as SLUG-21^20,41^ and SBN^58^ reveals that the removal of TcO_4_^−^/ReO_4_^−^ by SCP-IHEP-1 leads to little structural change of supramolecular framework, while anion removal by the latter ones rely on coordination of target anions with metal centers accompanied by irreversible significant structural arrangement (Supplementary Figure 24). Evidently, the inherent flexibility of soft supramolecular framework of SCP-IHEP-1 facilitates a fast dynamic recognition process, and thus enables superior kinetics as well as good reversibility of anion exchange. Especially, the involvement of CB8 macrocycles in SCP-IHEP-1 plays a vital role in directing supramolecular assembly process, and can be indeed an important contributor to high selectivity in terms of constructing ordered pores for anion trapping and interacting with trapped anions through a mass of hydrogen bonds. The fast kinetics, reversibility and selectivity as well as high efficiency by this type of soft supramolecular material make it outcompetes with traditional cationic MOM sorbents.

In order to understand the driving force underlying the specific ReO_4_^−^/TcO_4_^−^ uptake, theoretical calculation methods were used to analyze the interactions of CB8-based host cationic framework with different anions (NO_3_^−^, ReO_4_^−^, and TcO_4_^−^) within SCP-IHEP-1 or SCP-IHEP-1-Re. The GGA-PBE^59^ functional implemented in VASP 5.4^60^ was used to optimize the unit cells of the crystals, allowing relaxation of all the atom coordinates. With the fully optimized unit cells, three anion-containing tetrahedral pore models based on a simplified host system [**H**] consisting of the key components of the CB8 macrocycles were built (Supplementary Figure 25). Electrostatic potential (ESP) distribution analysis of these simplified models shows that, the portal carbonyls of CB8 exhibit negative ESP, while the waist CH_2_/CH groups exhibit positive ESP that facilitates interaction with negative-charged moieties such as NO_3_^−^ and ReO_4_^−^ (Supplementary Figure 26 and Supplementary Table 14). Besides the ESP maps, hydrogen bonding orbital interaction analysis from the MO perspective were also studied, and the results reveal that orbital interactions contribute to several hydrogen bonds as evidenced by that of [**H**] and ReO_4_^−^ (Supplementary Figure 27).

**Fig. 8:**
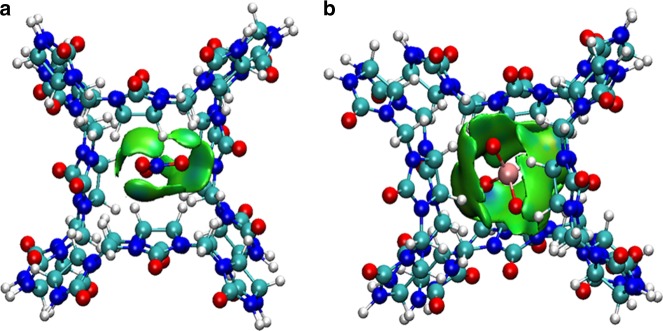
Intermolecular interactions (isosurfaces: 0.005 a.u.) for different models using IGM analysis. **a** CB8-based host [**H**] and NO_3_^−^; **b** CB8-based host [**H**] and ReO_4_^−^ (blue represents a strong attraction, and red denotes a strong repulsion). All isosurfaces are colored according to a BGR (blue-green-red) scheme over the electron density range −0.05 < sign(*λ*_2_)*ρ* < 0.05 a.u

To further clarify hydrogen bonding interactions between CB8-based host **[H]** and anions, quantum theory of atoms in molecules (QTAIM) analysis was performed at the B3LYP-D3(BJ)/6-311+G(d,p) level of theory (see Supplementary Methods for details). Several bond critical points (BCPs) between [**H**] and oxygen atoms of NO_3_^−^ and ReO_4_^−^ can be observed (Supplementary Figure 28), indicating noncovalent interactions between CB8-based host [**H**] and these anions. The parameters of electron density (*ρ*), Laplacian of electron density (∇^2^*ρ*), kinetic energy density (*G*), and potential energy density (*V*) at the representative BCPs are listed in Supplementary Table 15. These values are in the range 0.02 < *ρ* < 0.07 e/Å^3^, 0.2 < ∇^2^*ρ* < 0.8 e/Å^5^, 4.5 < *G* < 18.3 kJ/mol/Bohr^3^, −15.7 < *V* < -3.1 kJ/mol/Bohr^3^, respectively. For shorter H-Bond lengths (e.g., 2.511 Å), these values are within the scope of weak hydrogen bonding, while the larger ones belong to van der Waals interactions^61–63^. These intermolecular interactions can be further detected by independent gradient model (IGM)^64^ analysis and reduced density gradient (RDG)^65^ analysis (Fig. 8 and Supplementary Figure 29 and 30), and proved be weak hydrogen bonding (light-blue area in isosurfaces) and van der Waals interactions (green area in isosurfaces). The results are in excellent agreement with the QTAIM analysis. Besides, the low-density and low-gradient spikes of RDG analysis also confirm the presence of noncovalent interactions (Supplementary Figure 31). In all, the calculation results suggest the vital role of hydrogen bonds in stabilizing the [**H**]-anion inclusion motifs and thus facilitating the effective accommodation of target anions in the flexible cationic supramolecular framework.

We computed hydration energies, binding energies and total energies for anion exchange with [**H**] based on anion-containing tetrahedral pore models built as above (see Supplementary Figure 32). The computed total energies (BE_aq_) are similar for TcO_4_^−^ and ReO_4_^−^, and both of them are larger than that of NO_3_^−^-[**H**] model (Supplementary Table 16). Further energy analysis shows that difference of anion-[**H**] binding energies (ΔBE_gas_) for all these three models are not significant, whilst the difference of hydration energy (ΔE_hyd_) between ReO_4_^−^ and NO_3_^−^ is dominant. This result suggests the vital role of hydrophobic nature of CB8-based methylene/methylidyne-rich tetrahedral pores of SCP-IHEP-1 in selective TcO_4_^−^/ReO_4_^−^ removal, and thus provides a valuable evidence for the Hofmeister bias selectivity of SCP-IHEP-1 mentioned before.

## Discussion

We put forward a tactic using anion-adaptive cationic material with structural dynamics for ^99^TcO_4_^−^ sequestration. As a conceptual prototype, a soft supramolecular material, SCP-IHEP-1, was synthesized and has been demonstrated to exhibit excellent removal performance of TcO_4_^−^ (and ReO_4_^−^), especially in selectivity against competing anions. This exceptional performance is attributed to the anion-adaptive rearrangement of the CB8-surrounded pores, which can adapt to the encapsulated anionic guest. Hydration energy difference between displaced NO_3_^−^ and target oxoanions should be the essential driving force for facilitating selective TcO_4_^−^ uptake of sorbents with hydrophobic pores. The result suggests the potential of this anion-adaptive cationic material SCP-IHEP-1 for effective TcO_4_^−^ removal, and most importantly, paves a way for developing high efficiency sorbents for anion removal based on soft sorbent materials with anion-adaptive dynamics and efficient anion recognition capability to achieve selective and specific anion binding.

## Methods

### Materials

Caution! Tc-99 possesses significant health risks when inhaled or digested and should be handled according to standard precautions and procedures. All Tc-99 studies were conducted in a licensed laboratory dedicated to radiological investigations. Cucurbit[8]uril (CB8) was synthesized according to the previously-reported literature^66^. 4,4′-bipyridine (bpy), Cu(NO_3_)_2_, NH_4_ReO_4_, and other reagents were analytically pure and used as received. ^99^TcO_4_^−^ stock solutions were prepared by dissolving certain amounts of NH_4_TcO_4_ (99%) in deionized water or NO_3_^−^ solution as desired.

### One-pot synthesis of SCP-IHEP-1

An aliquot of 0.2 M Cu(NO_3_)_2_ aqueous solution (200 μL, 0.04 mmol) was added to a suspension of 4,4′-bipyridine (bpy) (0.006 g, 0.04 mmol) and CB8 (0.026 g, 0.02 mmol) in water (2 mL) in a stainless-steel vessel. The mixture was sealed, and kept at 150 °C for 48 h. After cooling to room temperature, the obtained blue microcrystals of SCP-IHEP-1 were filtered, rinsed with water and ethanol three times, and dried in air at room temperature. Yield: 0.026 g (59% based on CB8).

### Two-step synthesis of SCP-IHEP-1

(a) 2bpy@CB8: 4,4′-bipyridine (bpy) (0.006 g, 0.04 mmol) was added to a suspension of CB8 (0.026 g, 0.02 mmol) in water (2 mL). After incubation in a stainless-steel vessel at 150 °C for 24 h, colorless block crystals of 2bpy@CB8 were obtained in a quantitative yield during cooling to room temperature. The structure of 2bpy@CB8 was confirmed by single-crystal structure determination as [(bpy)_2_@CB8]_0.5_·[(bpy)_2_@CB8]_0.5_·19H_2_O (Figure S4) and ^1^H-NMR spectra (Figure S5). ^1^H NMR (500 MHz, D_2_O, δ ppm): 8.49 (m, 8H); 7.42 (m, 8H), 5.84 (d, 16H), 5.52 (s, 16H), 4.22 (d, 16H). (b) SCP-IHEP-1: 0.2 M Cu(NO_3_)_2_ aqueous solution (500 μL, 0.04 mmol) was added to a suspension of 2bpy@CB8 intermediate obtained as described above in water (2 mL) in a stainless-steel vessel. The mixture was sealed, and kept at 150 °C for 48 h. After cooling to room temperature, light-blue block crystals of SCP-IHEP-1 were obtained in a high yield (~0.045 g, >99%).

### Synthesis of Cu-bpy

An aliquot of 0.2 M Cu(NO_3_)_2_ aqueous solution (500 μL, 0.10 mmol) was added to a suspension of 4,4′-bipyridine (bpy) (0.016 g, 0.10 mmol) in water (1.5 mL) in a stainless-steel vessel. The mixture was sealed, and heated slowly to 150 °C in a period of 24 h, and kept at 150 °C for another 48 h. After slowly cooling to room temperature in a period of 24 h, the obtained blue regular plate-like crystals of Cu-bpy were filtered, rinsed with water three times, and dried in air at room temperature. Yield: 0.013 g.

### Synthesis of SCP-IHEP-1-Re

To elucidate the exchange and recognition mechanism for SCP-IHEP-1 with ReO_4_^−^ (and by inference TcO_4_^−^), crystals of ReO_4_^−^-incorporated SCP-IHEP-1 sorbent were synthesized through an in situ assembly method. The detailed synthesis procedure is as follows: 0.2 M Cu(NO_3_)_2_ aqueous solution (200 μL, 0.04 mmol) and 0.2 M NH_4_ReO_4_ aqueous solution (400 μL, 0.04 mmol) was added to a suspension of 2bpy@CB8 obtained as described above in water (2 mL) in a stainless-steel vessel. The mixture was sealed, and kept at 150 °C for 48 h. After cooling to room temperature, small light-blue prismatic crystals of SCP-IHEP-1-Re were obtained. The PXRD pattern of SCP-IHEP-1 after ReO_4_^−^ uptake collected is fully consistent with the simulated PXRD pattern based on crystal data for SCP-IHEP-1-Re obtained above, suggesting identical structures.

### Synthesis of Cu-bpy-Re

An aliquot of 0.2 M Cu(NO_3_)_2_ aqueous solution (500 μL, 0.10 mmol) and 0.2 M NH_4_ReO_4_ aqueous solution (400 μL, 0.04 mmol) was added to a suspension of 4,4′-bipyridine (bpy) (0.006 g, 0.04 mmol) in water (2.0 mL) in a stainless-steel vessel. The mixture was sealed, and heated slowly to 150 °C in a period of 24 h, and kept at 150 °C for another 48 h. Blue block crystals of Cu-bpy-Re were obtained after slowly cooling to room temperature in a period of 24 h.

### X-ray single-crystal structure determination

X-ray diffraction data for SCP-IHEP-1, Cu-bpy, and Cu-bpy-Re were acquired on a Bruker D8 VENTURE X-ray CMOS diffractometer with a Cu Kα X-ray source (*λ* = 1.54178 Å) at room temperature. Data frames were collected using the program APEX 3 and processed using the program SAINT routine in APEX 3. Data collection for 2bpy@CB8 and SCP-IHEP-1-Re was acquired with synchrotron radiation at Beijing Synchrotron Radiation Facility (BSRF, *λ* = 0.72 Å) using a MAR CCD detector. The crystal was mounted in nylon loops and cooled in a cold nitrogen-gas stream at 100 K. Data were indexed, integrated and scaled using DENZO and SCALEPACK from the HKL program suite. All crystal structures were solved by means of direct methods and refined with full-matrix least squares on SHELXL-97^67^, and refined with full-matrix least squares on SHELXL-2014^67,68^. The crystal data of all compounds are given in Supplementary Table 1.

### Batch experiments

All the sorption experiments were conducted using the batch sorption method. The solid/liquid ratio performed in all batch experiments was 0.5 g L^−1^. In a typical experiment, 4 mg of SCP-IHEP-1 or Cu-bpy was added into 8 mL of aqueous solution with a certain concentration of ReO_4_^−^. The pH values of the solutions were adjusted as required using NaOH and HNO_3_ and were measured on a digital pH-meter. The mixture was stirred for a specified time (*t*, min) at a specified temperature (*T*, K), and separated with a 0.22 μm nylon membrane filter. The concentrations of ReO_4_^−^ in aqueous solution were determined by inductively coupled plasma optical emission spectrometry (ICP-OES, Horiba JY2000-2).

## Supplementary information

Supplementary Information

## Data Availability

Details for measurements and characterization, detailed experimental procedures, computational methods are given in the Supplementary Methods. The X-ray crystallographic coordinates for structures reported in this study have been deposited in the Cambridge Crystallographic Data Center under accession numbers CCDC: 1874188 (2bpy@CB8), 1874189 (SCP-IHEP-1), 1874190 (SCP-IHEP-1-Re), 1894900 (Cu-bpy), and 1894899 (Cu-bpy-Re), respectively. These data can be obtained free of charge via http://www.ccdc.cam.ac.uk/data_request/cif). All data are either provided in the Article and its Supplementary Information or available from the corresponding author upon request.
