# Supplementary material for: Anion-adaptive crystalline cationic material for 99TcO4− trapping
Source: Nat Commun. 2019 Apr 4;10:1532. doi: 10.1038/s41467-019-09504-3 (PMC6449352; doi:10.1038/s41467-019-09504-3)
Supplement: Supplementary file 1 — Supplementary Information [file 41467_2019_9504_MOESM1_ESM.pdf]

Supplementary Information

Anion-Adaptive Crystalline Cationic Material for $^{99}TcO_4^-$ Trapping

Mei et al.

Supplementary Methods

General Methods for Property Characterization. ^{1}H-NMR spectra were recorded on a Bruker AVANCE III (500 MHz, Bruker, Switzerland) with deuterium oxide as solvent. Powder X-ray diffraction measurements (PXRD) were recorded on a Bruker D8 Advance diffractometer with Cu Kα radiation (λ = 1.5406 Å) in the range 5-50° with a step size of 0.02°. Morphologies and chemical compositions of sorbent samples were measured using a Hitachi S-4800 field-emission scanning electron microscope (SEM) equipped with Horiba 7593-H energy-dispersive X-ray spectroscopy (EDS). Thermogravimetric analysis (TGA) was recorded from a TA Q500 analyzer over the temperature range of 25-800 ℃ in air atmosphere with a heating rate of 5 ℃/min. Fourier transform infrared (IR) spectra were recorded from KBr pellets in the range of 4000-400 cm^{-1} on a Bruker Tensor 27 spectrometer. Solution concentrations of ReO_4^- ion were determined using an inductively coupled plasma optical emission spectrograph (ICP-OES, Horiba JY2000-2); concentrations of $^{99}TcO_4^-$ were determined by liquid scintillation counting (Perkin Elmer Quantulus 1220).

Computational Methods. For the energy analysis of formation of BPY-CB8, DFT calculations were performed with the BP86 functional[1] implemented in Gaussian 16 program[2]. For light atoms (H, C, N and O), the polarized all-electron 6-31+G(d) basis set was used for geometry optimizations and energy calculations. This approach previously provided reliable results for uranyl complexes.[3-7] Different models (2bpy@CB8 model based on BPY-CB8, SCP-IHEP-1 and SCP-IHEP-1-Re) and/or processing methods (unrestricted structure optimization and N(bpy)-fixed structure optimization) were used to evaluate the changes of Gibbs free energies (ΔG), enthalpies (ΔH) and entropies (ΔS) for the reactions between bpy and CB8 to form a bpy dimer within the CB8 cavity as shown in Supplementary Figure 6. The calculated results for ΔG, ΔH and ΔS are listed in Supplementary Table 2.

Density functional theory calculations were also used to study anion exchange mechanism and interactions of three anions (NO_3^-, ReO_4^- and TcO_4^-) with host materials. To gain accurate binding modes of these anions in their frameworks, the GGA-PBE[8] functional implemented in VASP 5.4[9] was used to optimize the unit cells of the crystals (considering the chemical similarity of TcO_4^- and ReO_4^-, the pseudo model for TcO_4^- interaction with the framework was obtained by replacing ReO_4^- with TcO_4^-), allowing relaxation of all the atom coordinates. The plane-wave cutoff of 400 eV and the Gaussian electron smearing method with $\sigma = 0.05$ eV were used here. During all the simulations, the Re $5d^5 6s^2$ and Tc $4d^5 5s^2$ valence electrons were treated explicitly.

With the fully optimized unit cells, three anion-containing tetrahedral pore models based on a simplified host system consisting of the key components of the CB8 macrocycles were built (the dangling bonds were saturated with H atoms) were built correspondingly to study the interactions of three anions (NO_3^-, ReO_4^- and TcO_4^-) with host materials with the Gaussian 16 package[2]. The Stuttgart energy-consistent quasi-relativistic small-core pseudopotentials ECP60MWB[10] were used for Re and Tc along with the corresponding valence basis sets ECP60MWB-SEG[10, 11]. For light atoms, the 6-311G(d, p) basis set was utilized for property predictions. The electrostatic potential (ESP) map was computed for quantitative molecular surface analysis with the aid of Multiwfn 3.5[12]. The Bader charge analysis[13] on the basicity of oxygen atoms of the anions was performed based on total charge density which includes both valence charge density and core charge density, and the results were compared to those calculated by using natural population analysis (NPA), and Mulliken charges, respectively.

To demonstrate the interaction between the framework and the anion, quantum theory of atoms in molecules (QTAIM) analysis[13-16] was performed with the optimized model fragments of

SCP-IHEP-1 and SCP-IHEP-1-Re at the B3LYP-D3(BJ)/6-311+ G(d, p) level of theory. The calculations of noncovalent interactions between the cationic framework and the anion were performed by the B3LYP[17, 18] method with Grimme's D3 dispersion correction[19] based on the simplified anion-containing model fragments. For Re, scalar relativistic effects were considered by the quasi-relativistic effective core potentials ECP60MWB and ECP60MWB-SEG valence basis sets[20-22], and the 6-311+G(d,p) basis sets were used for light atoms H, C, N, O. The quantum theory of atoms in molecules (QTAIM)[13-16] analysis, independent gradient model (IGM)[23] analysis, and reduced density gradient (RDG)[24] analysis were carried out with the Multiwfn 3.5 program[25]. Molecular plots were visualized by the VMD 1.9.2 program[26].

Energy analysis was performed with the widely used functional, BP86[1]. The binding energy of these anions with the framework was calculated as the sum of electronic energy and solvation energy. Solvent effects were evaluated by using the conductor-like polarizable continuum model (CPCM) [27, 28] and the Klamt atomic radii [29] based on default parameters. Binding energy (BE_{gas} and BE_{aq}) between the host framework ([**H**]) and different anions in different environments ('*dehyd*' means dehydrated and '*aq*' means hydration in aqueous solution) are defined as follows (Supplementary Eq. 1-4):

$$[H] + NO_3^-(dehyd) \xleftrightarrow{\mathrm{BE}_{gas}(NO_3^-)} NO_3^- \in [H] \tag{1}$$

$$[H] + AO_4^-(dehyd) \xleftrightarrow{\mathrm{BE}_{gas}(AO_4^-)} AO_4^- \in [H] \quad (A = Tc, Re) \tag{2}$$

$$[H] + NO_3^-(aq) \xleftrightarrow{\mathrm{BE}_{aq}(NO_3^-)} NO_3^- \in [H] \tag{3}$$

$$[H] + AO_4^-(aq) \xleftrightarrow{\mathrm{BE}_{aq}(AO_4^-)} AO_4^- \in [H] \quad (A = Tc, Re) \tag{4}$$

The total reaction energy after NO_3^- exchange by TcO_4^-/ReO_4^-, $\Delta BE_{aq}(NO_3^- \text{-} AO_4^-)$, could be calculated according reaction paths as shown in Supplementary Figure 32 (E_{hyd} means hydration energy). All the calculated data are listed in Supplementary Table 14.

Equations Used for Batch Experiments. All the sorption experiments were conducted using the batch sorption method and the removal efficiency, R_m (%), sorption capacity, q_e (mg g^{-1}) and distribution coefficient, K_d (mL g^{-1}), were calculated by the following equations:

$$R_m = \frac{c_0 - c_e}{c_0} \times 100\% \tag{5}$$

$$q_e = \frac{c_0 - c_e}{m} \times V \tag{6}$$

$$K_d = \frac{c_0 - c_e}{c_e} \times \frac{V}{m} \tag{7}$$

where c_0 (mg g^{-1}) and c_e (mg L^{-1}) are the initial and equilibrium concentration of TcO_4^-/ReO_4^-, and V is the total volume of solution, m is the mass of the sorbent.

Sorption Data Fitting by Kinetics Models. In order to elaborate the sorption kinetic of TcO_4^-/ReO_4^-, pseudo-first-order kinetic model and pseudo-second-order kinetic model were used to analyze the experimental kinetic data. The linearized forms of the two models are given as follows:

$$\log(q_e - q_t) = \log q_e - \frac{k_1 t}{2.313} \tag{8}$$

$$\frac{t}{q_t} = \frac{1}{k_2 q_e^2} + \frac{t}{q_e} \tag{9}$$

where q_e (mg g^{-1}) and q_t (mg g^{-1}) are the sorption capacity of sorbed TcO_4^-/ReO_4^- at equilibrium and at time t respectively, and k_1 (min^{-1}) and k_2 (g mg^{-1} min^{-1}) are the pseudo-first-order and pseudo-second-order sorption rate constants, respectively. The plots of $\log(q_e$-$q_t)$ versus t and t/q_t versus t both give straight lines, and k_1 and k_2 as well as q_e can be calculated from the slope and intercept. The model parameters and the correlation coefficient obtained by both the models for exchange kinetics of SCP-IHEP-1 and Cu-bpy with ReO_4^- are listed in Supplementary Table 5 and Table 6, respectively.

Sorption Data Fitting by Isotherm Models. Two models, the Langmuir model and the Freundlich equation, are used to clarify the sorption process. The Langmuir model assumes that the sorption of target ions occurs on a homogenous surface by monolayer sorption and there are no interaction between adsorbed ions, with homogeneous binding sites and equivalent sorption energies. The Freundlich equation is an empirical equation based on sorption on a heterogeneous surface, of which the isotherm assumes that adsorbent surface sites have a spectrum of different binding energies. The linear equations forthe Langmuir isotherm model and the Freundlich equation are expressed as follows:

$$\frac{c_e}{q_e} = \frac{1}{k_L q_m} + \frac{c_e}{q_m} \quad (10)$$

$$\ln q_e = \ln k_F + \frac{1}{n} lnc_e \quad (11)$$

where q_e (mg g^{-1}) and c_e (mg L^{-1}) are the sorption capacity and concentration of sorbed TcO_4^-/ReO_4^- at equilibrium starting from different initial concentrations. q_m is the maximum sorption capacity corresponding to complete monolayer coverage (mg g^{-1}). k_L is a constant indirectly related to sorption capacity and energy of sorption (L mg^{-1}), which characterizes the affinity of the adsorbate and adsorbent, while k_F and n are the Freundlich constants related to the sorption capacity and the sorption intensity, respectively. The plots of c_e/q_e vs c_e and lnq_e vs lnc_e both give straight lines, and q_m and k_L as well as k_F and n could be calculated from the slope and intercept. The model parameters and the correlation coefficient obtained by both the models for the sorption isotherm study of SCP-IHEP-1 with ReO_4^- are listed in Supplementary Table 7 and Table 9, respectively.

Thermodynamic Parameters Fitting. The Van’t Hoff equation was used to analyze the experimentally observed effect of temperature on sorption. The linearized form of Van’t Hoff equation is given as follows:

$$\ln K_d = \frac{\Delta S}{R} - \frac{\Delta H}{T} \quad (12)$$

R (J mol^{-1} K^{-1}) is the ideal gas constant, and K_d is the distribution coefficient at temperature T. The thermodynamic parameters, enthalpy change (ΔH, kJ mol^{-1}) and entropy change (ΔS, J mol^{-1}),

were calculated using the linear form of the Van't Hoff equation. The Gibbs free energy change (ΔG, kJ mol^{-1}) was determined by the following equation:

$$\Delta G = \Delta H - \mathrm{T}\Delta S \quad (13)$$

All thermodynamic parameters obtained by the Van't Hoff equation and the corresponding Gibbs free energy change are listed in Supplementary Table 10.

Exchange Kinetics Studies. The exchange kinetics experiments of SCP-IHEP-1 or Cu-bpy towards ReO_4^- were determined by monitoring the concentration of ReO_4^- as a function of time. In detail, 20 mg of SCP-IHEP-1 sorbent was added into 40 mL of an aqueous solution of ReO_4^- (SCP-IHEP-1, ~45 mg L^{-1}; SCP-IHEP-1, ~38 mg L^{-1}). The mixture was stirred for a desired contact time (from 1 min to 8 hours) at pH 7, and then separated using a 0.22 μm nylon membrane filter. The concentrations of ReO_4^- in aqueous solution were determined by ICP-OES.

Sorption Isotherm Experiments. The sorption isotherm experiments of SCP-IHEP-1 or Cu-bpy towards ReO_4^- were determined by varying the concentration of ReO_4^- ranging from 20 to 200 mg L^{-1}. In a typical proecss, 4 mg of sorbents were added into 8 mL of aqueous solution with certain concentration of ReO_4^-. After stirring for 12 hours at pH 7 to ensure the equilibrium, the mixture was then separated using a 0.22 μm nylon membrane filter. The concentrations of ReO_4^- in aqueous solution were determined by ICP-OES.

Exchange Reversibility Studies and Reusability Studies. The SCP-IHEP-1 material sorbed with 45 ppm of ReO_4^- (4 mg sorbent/8 mL aqueous solution of ReO_4^-) was added into a desorption solution containing 0.5 M $NaNO_3$. The resulting mixture was stirred for 12 hours, followed by the determination of the concentrations of ReO_4^- in aqueous solution by ICP-OES. The recovered material was subject to the recyclability experiments at the solid-liquid ratio of 0.5 g/L using ReO_4^- solution (45 mg L^{-1}) at pH 7.

Effect of pH Studies. The effect of pH on the ReO_4^- removal was evaluated by varying the pH of aqueous solution ranging from 2 to 10. The pH of the solution was adjusted as required using

NaOH and HNO_3 and was measured on a digital pH-meter. After stirring for 12 hours, the mixture was separated with a 0.22 μm nylon membrane filter and the concentrations of ReO_4^- after sorption in aqueous solution were determined by ICP-OES.

Anion Exchange Selectivity Studies. The effect of other competing anions including NO_3^-, SO_4^{2-}, CO_3^{2-}, PO_4^{3-}, and ClO_4^- on the sorption of ReO_4^- by SCP-IHEP-1 was investigated by adding 0.24 mM $NaNO_3$, Na_2SO_4, Na_2CO_3, NaH_2PO_4, and $NaClO_4$ solutions receptively into a 0.24 mM ReO_4^- solution at pH 7. The effect of excess NO_3^- was performed by adding 0.24 mM, 1.2 mM, 2.4 mM, 4.8 mM, or 24 mM $NaNO_3$ solutions respectively into a 0.24 mM ReO_4^- solution at pH 7. The effect of SO_4^{2-} was performed by adding 0.24 mM, 2.4 mM, 24 mM, 240 mM, or 960 mM Na_2SO_4 solutions respectively into a 0.24 mM ReO_4^- solution.

Exchange Experiments Using a Simplified Simulated Waste Solution. The ion exchange experiment of SCP-IHEP-1 towards $^{99}TcO_4^-$ was directly tested using a simplified simulated waste solution containing 9.8 ppm of $^{99}TcO_4^-$ in 0.03 M HNO_3 (the initial concentration of NO_3^- is ~500 times higher than that of $^{99}TcO_4^-$). The exchange kinetics was monitored as a function of time. In detail, 20.5 mg of SCP-IHEP-1 sorbent was added into 40 mL of an acidic aqueous solution of $^{99}TcO_4^-$ (9.8 mg L^{-1} in 0.03 M HNO_3). After the pH of the solution was adjusted to 6.87 using NaOH, the mixture was stirred for a desired contact time (from 1 min to 4 hours) by a magnetic bar, and then separated using a 0.22 μm nylon membrane filter. The concentrations of $^{99}TcO_4^-$ in aqueous solution were measured using a liquid scintillation counting system (Perkin Elmer Quantulus 1220).

Effect of temperature and thermodynamic studies. The effect of temperature on the sorption of ReO_4^- by SCP-IHEP-1 was investigated at 300, 305, 310, 315 and 320 K. The sorption amount of ReO_4^- gradually decreased (approximately 30 mg L^{-1}) with the increase in environmental temperature from 300 to 320 K, indicating that the present sorption may be an exothermic process.

Hydrolytic Stability Measurements. Hydrolytic stability measurements were studied by

exposure of SCP-IHEP-1 crystals in HNO_3 or NaOH of different pH followed by shaking vigorously in an oscillator for 12 h at 298 K. The PXRD results demonstrate that SCP-IHEP-1 is stable in aqueous solutions within pH range from 3 to 11.

Supplementary Figures

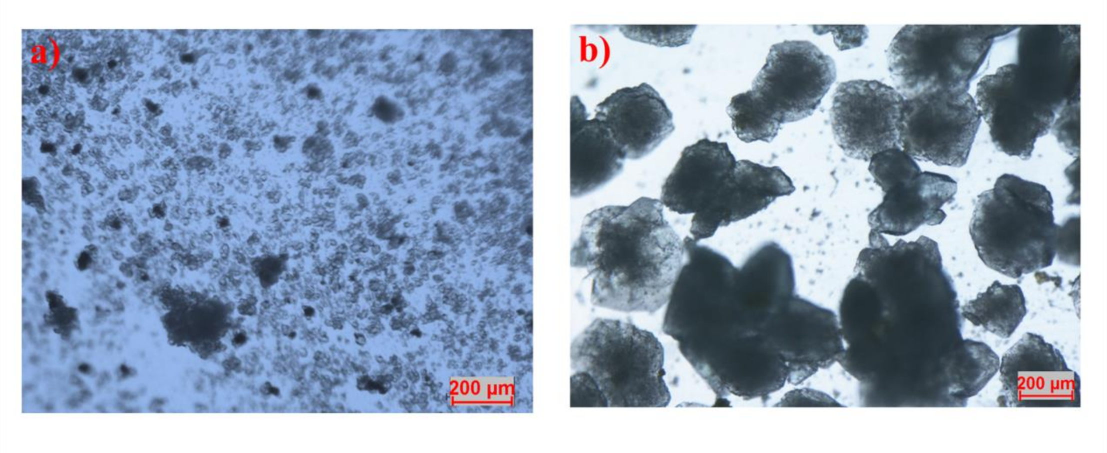

Supplementary Figure 1. Crystals of SCP-IHEP-1: (a) Optical photograph of microcrystals obtained through multi-component one-pot synthesis (scale bar: 200 μm); (b) optical photograph of block crystals obtained through two-step synthesis using pre-organized BPY-CB8 intermediates as the ligand (scale bar: 200 μm).

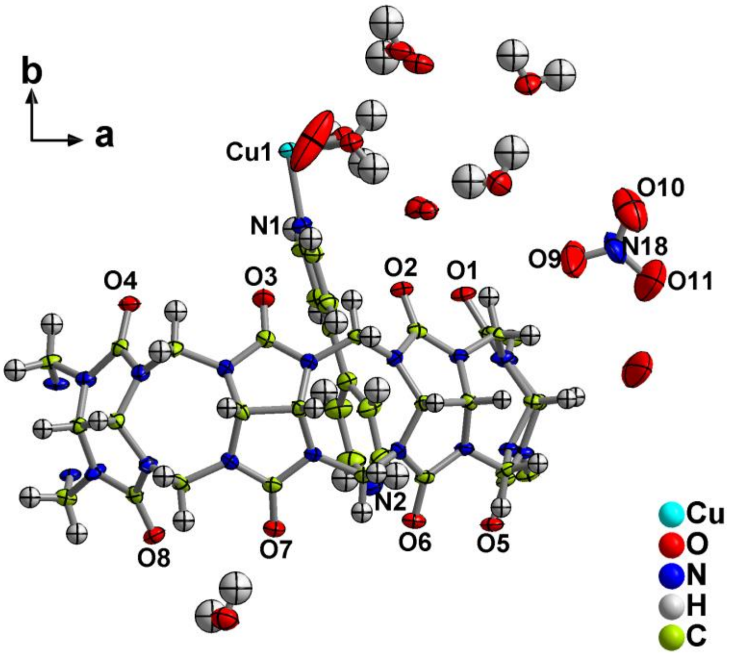

Supplementary Figure 2. ORTEP drawing of the asymmetric unit in the crystal structure of SCP-IHEP-1 with the 50% probability level for thermal ellipsoids.

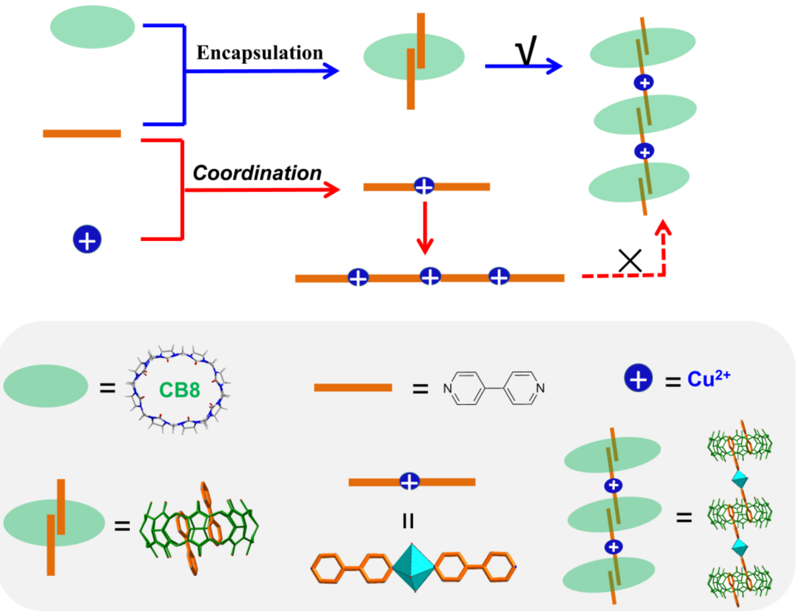

Supplementary Figure 3. Possible competition between metal coordination and supramolecular encapsulation for bpy during synthesis of SCP-IHEP-1. The proposed assembly process indicated by a check mark is firstly supramolecular encapsulation of CB8 followed by the assembly via bpy-Cu^{2+} coordination to form the final 1D chain.

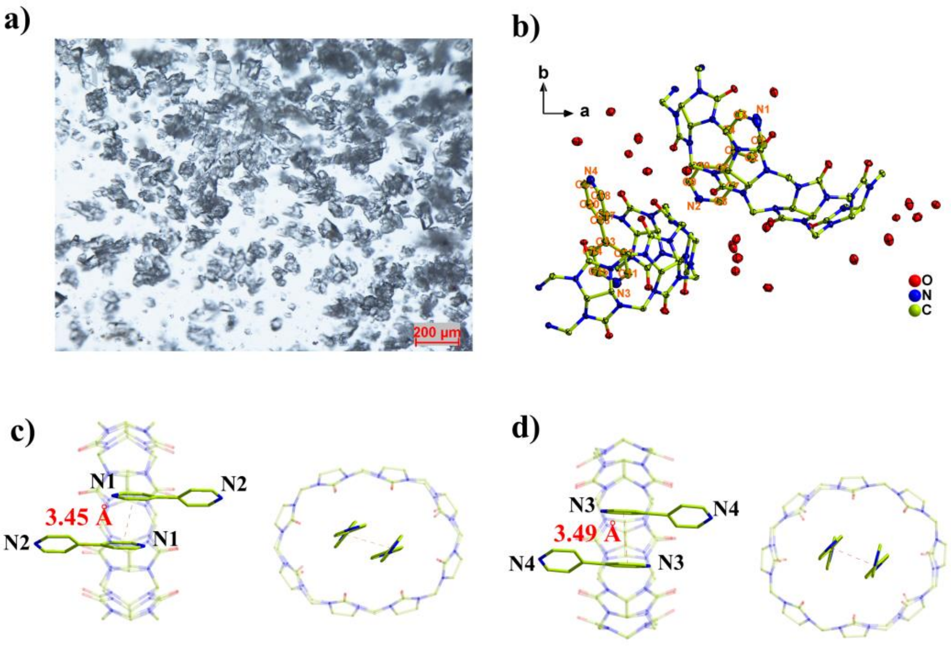

Supplementary Figure 4. Crystal structure of 2bpy@CB8: a) Optical photograph of small crystals of 2bpy@CB8 ($[(bpy)_2@CB8]_{0.5}$ $\{(bpy)_2@CB8]_{0.5}$ $\cdot 19H_2O$); b) the asymmetric unit in the crystal structure of 2bpy@CB8; c-d) two pi-pi stacking patterns (~3.45 Å and 3.49 Å) observed for bpy dimer in CB8, which differ from that found in SCP-IHEP-1 to reveal the structural variability of the flexible CB8-based 2bpy@CB8 encapsulation motif.

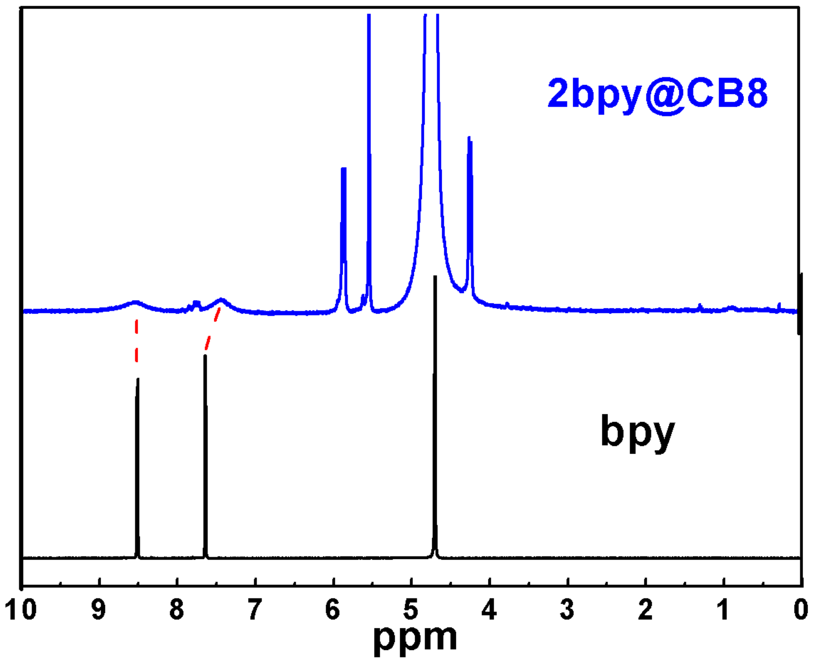

Supplementary Figure 5. ^{1}H NMR of 2bpy@CB8 dissolved in D_2O showing signals of 2bpy@CB8 motif with neat bpy for comparison. The fused signals for encapsulated and non-encapsulated bpy reveal the encapsulation and release of bpy in CB8 in the aqueous solution (D_2O) to be a fast exchange process.

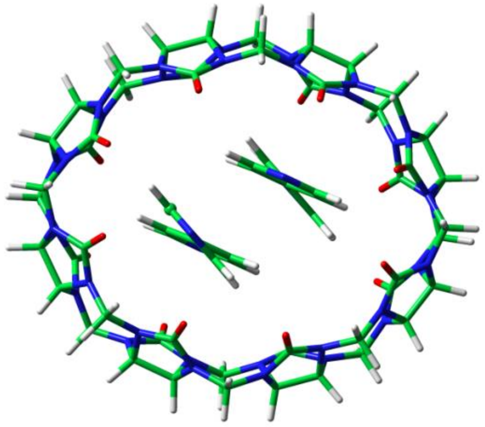

Supplementary Figure 6. Basic model of BPY-CB8 used to evaluate changes in Gibbs free energies (ΔG), enthalpies (ΔH) and entropies (ΔS) for reactions between bpy and CB8 to form a bpy dimer in the CB8.

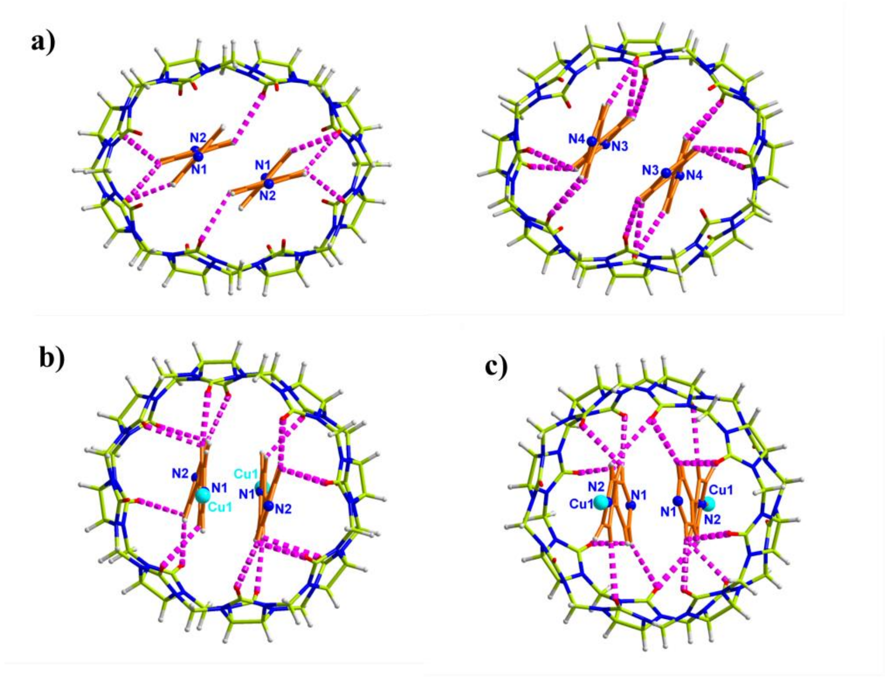

Supplementary Figure 7. Hydrogen bonds between CB8 and bpy molecules for different compounds: (a) 2bpy@CB8 with two different 2bpy@CB8 motifs; (b) SCP-IHEP-1 and (c) SCP-IHEP-1-Re.

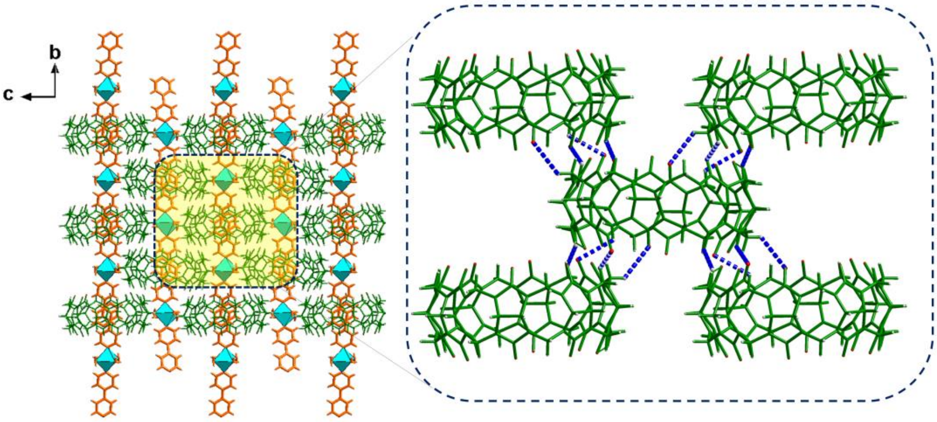

Supplementary Figure 8. Interchain hydrogen bonds for the construction of SCP-IHEP-1: (left) the three-dimensional cationic supramolecular framework of SCP-IHEP-1 cross-linked by interchain hydrogen bonds; CB8 macrocycles are in green, bpy molecules are in orange and Cu coordination spheres are in light blue; (right) hydrogen bonds between adjacent CB8 molecules ($d_{H\cdot\cdot O}$(C21-H21B ··O5), 3.050 (3) Å; $d_{H\cdot\cdot O}$(C27-H27B ··O4), 3.002(4)Å; $d_{H\cdot\cdot O}$(C28-H28B ··O4), 2.645(4)Å; $d_{H\cdot\cdot O}$(C16-H16B ··O2), 2.733(3) Å). Hydrogen bonds are indicated by dark blue dashed lines, and the bpy-Cu motifs have been omitted for clarity.

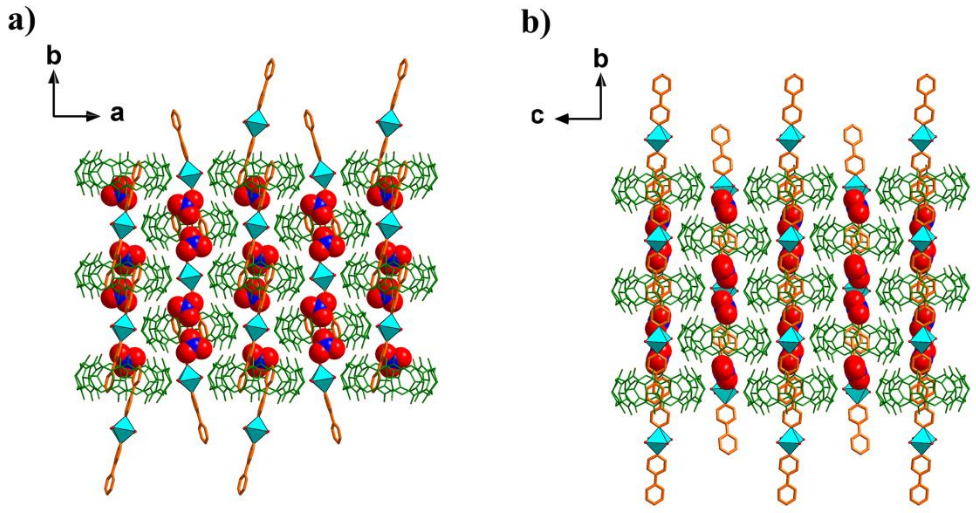

Supplementary Figure 9. Nitrate-incorporated three-dimensional cationic supramolecular framework of SCP-IHEP-1: (a) 3D stacking viewed from axis *c*; (b) 3D stacking viewed from axis *a*. Color codes: space-filling models with red and blue balls denote nitrate ions; CB8 macrocycles are in green, bpy molecules are in orange and Cu coordination spheres are in light blue.

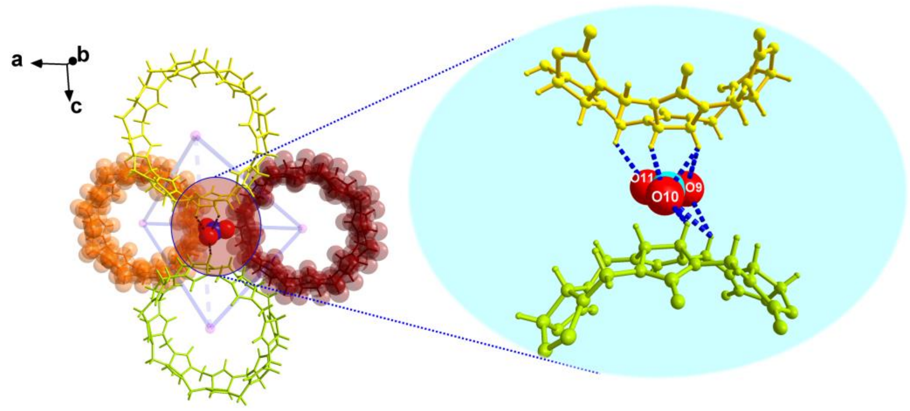

Supplementary Figure 10. A nitrate ion in a tetrahedral cavity formed by three CB8 interacts with only two CB8 through a set of hydrogen bonds (insert: an enlarged view of hydrogen bond networks around the nitrate): $d_{H\cdot\cdot O}$ (C21-H21B…O11) = 3.079(14) Å, $d_{H\cdot\cdot O}$ (C25-H25…O10)= 2.672(8) Å, $d_{H\cdot\cdot O}$ (C25’-H25’…O10) = 2.414(8) Å, $d_{H\cdot\cdot O}$ (C22-H22A…O10) = 3.074(8) Å, $d_{H\cdot\cdot O}$ (C24-H24…O10) = 2.941(7) Å, $d_{H\cdot\cdot O}$ (C22-H22A…O9) = 3.038(12) Å, $d_{H\cdot\cdot O}$ (C24-H24…O9) = 3.117(10) Å.

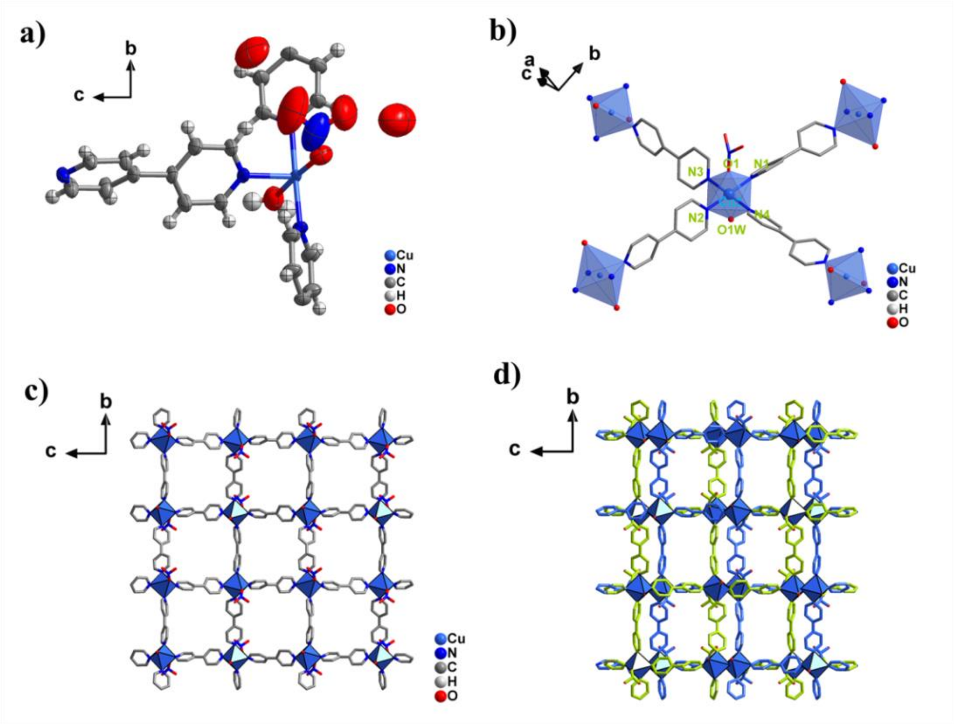

Supplementary Figure 11. Crystal structure of Cu-bpy: a) ORTEP drawing of asymmetric unit with the 50% probability level for thermal ellipsoids; b) coordination sphere of six-coordinated Cu^{2+} node with deformed octahedral geometry; c) 3D framework of Cu-bpy viewed from axis *a*; d) final crystal packing mode based on two-fold interpenetrating 3D frameworks.

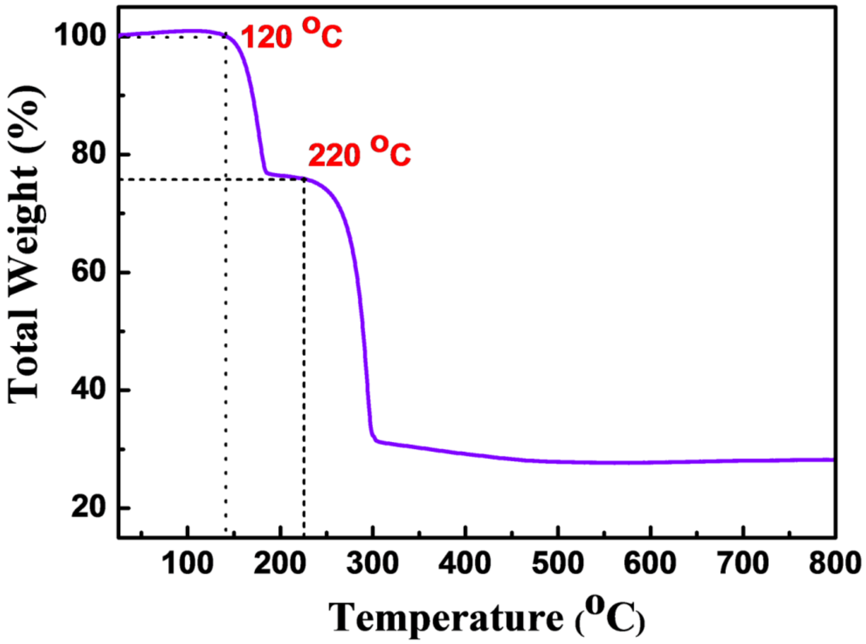

Supplementary Figure 12. Thermogravimetric analysis (TGA) in air for Cu-bpy: dehydration temperature, ~120 ℃; skeletal collapse temperature, ~220 ℃.

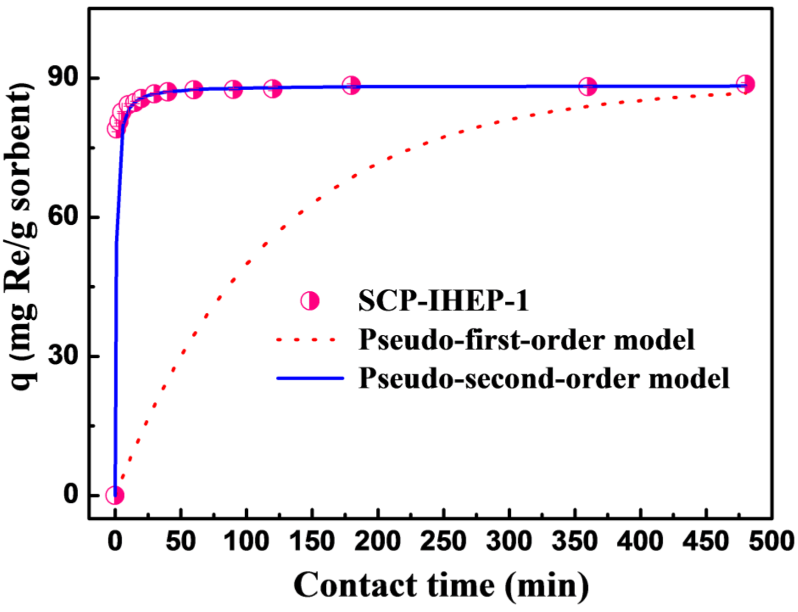

Supplementary Figure 13. The removal of ReO_4^- by SCP-IHEP-1 as a function of contact time (c_0 = 44.6 mg L^{-1}, V = 40 mL, m = 20 mg and pH = 6.96; half-filling pink dot: experimental data; blue solid line: the fitting result for pseudo-second-order model; red dash line: the fitting result for pseudo-first-order model). Error bars represent the s.d. of uncertainty for each point.

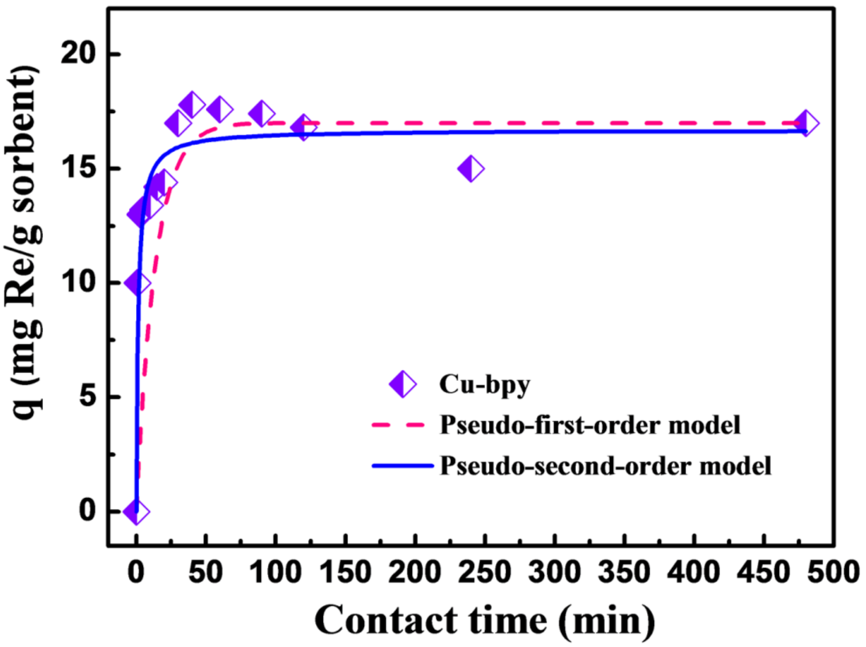

Supplementary Figure 14. The removal of ReO_4^- by Cu-bpy as a function of contact time (c_0 = 37.3 mg L^{-1}, V = 40 mL, m = 20 mg; half-filling purple diamond: experimental data; blue solid line: the fitting result for pseudo-second-order model; red dash line: the fitting result for pseudo-first-order model).

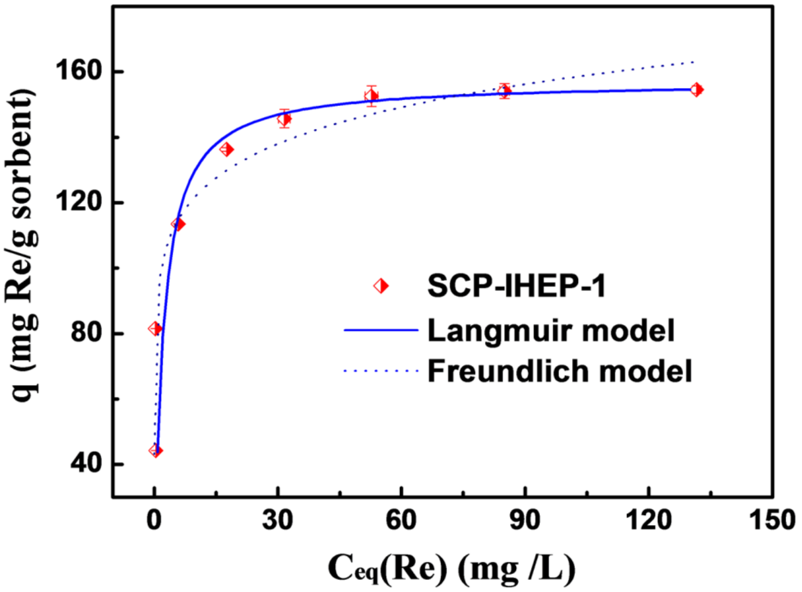

Supplementary Figure 15. Sorption isotherms of SCP-IHEP-1 with sorption capacity as a function of equilibrium concentration of ReO_4^- (T = 300 K, t = 12 h, V = 8 mL, m = 4 mg and pH = 7.03; half-filling red diamond: experimental data; blue solid line: the fitting result for Langmuir model; blue dash line: the fitting result for Freundlich equation). Error bars represent the s.d. of uncertainty for each point.

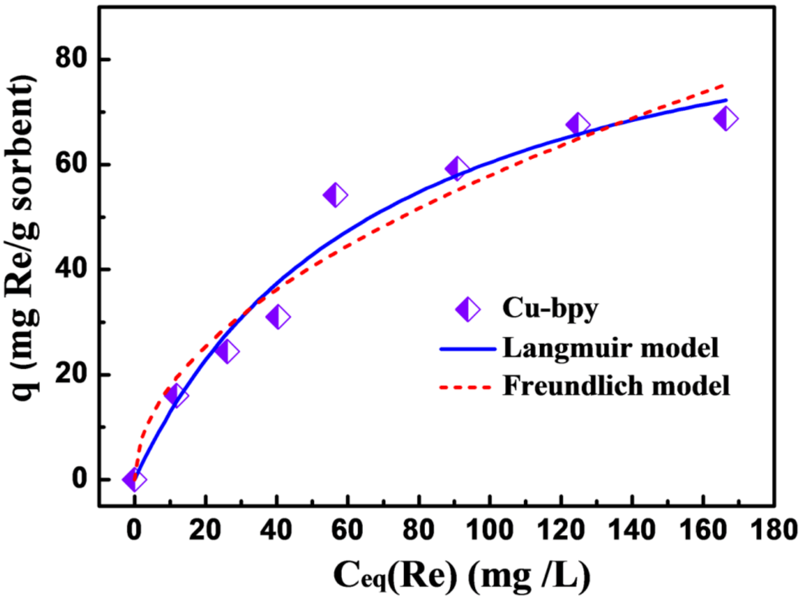

Supplementary Figure 16. Sorption isotherms of Cu-bpy with sorption capacity as a function of equilibrium concentration of ReO_4^- (T = 300 K, t = 12 h, V = 8 mL, m = 4 mg; half-filling red diamond: experimental data; blue solid line: the fitting result for Langmuir model; blue dash line: the fitting result for Freundlich equation).

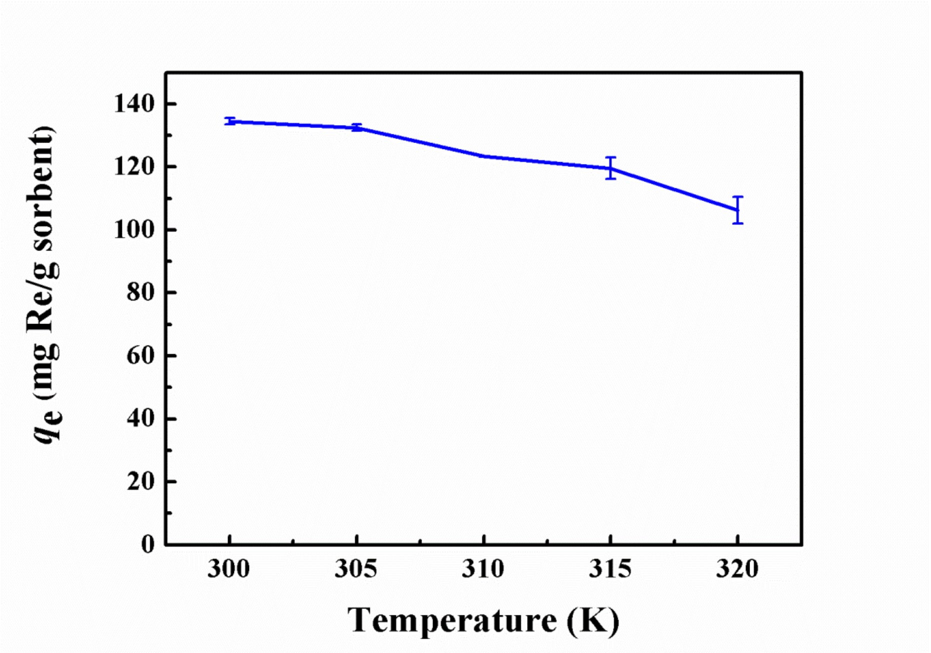

Supplementary Figure 17. Effect of temperature on the removal of ReO_4^- by SCP-IHEP-1 (c_0 = 94.4 mg L^{-1}, t = 12 h, V = 8 mL, m = 4 mg and pH = 7.21). Error bars represent the s.d. of uncertainty for each point.

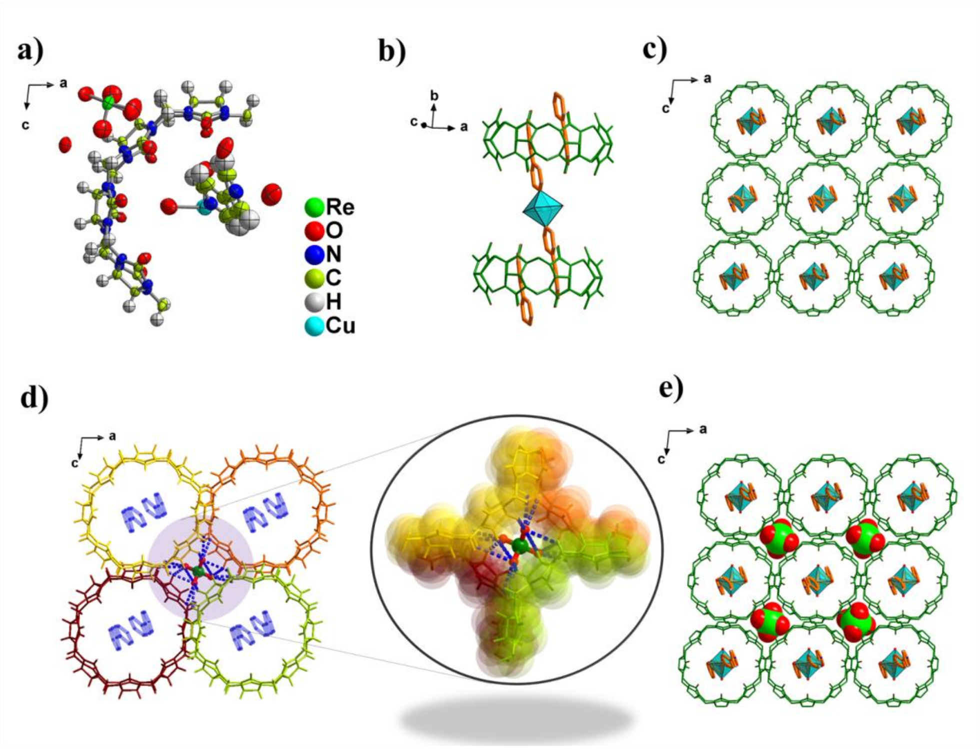

Supplementary Figure 18. Crystal structure of SCP-IHEP-1-Re: a) ORTEP drawing of asymmetric unit with the 50% probability level for thermal ellipsoids; b) main backbone of SCP-IHEP-1-Re consisting of bpy-CB8 encapsulation motif linked by Cu^{2+} coordination; c) 3D cationic supramolecular framework of SCP-IHEP-1-Re viewed from *b* axis; d-e) ReO_4^- trapped in a CB8-based tetragonal cavity; f) 3D supramolecular framework filled with ReO_4^-.

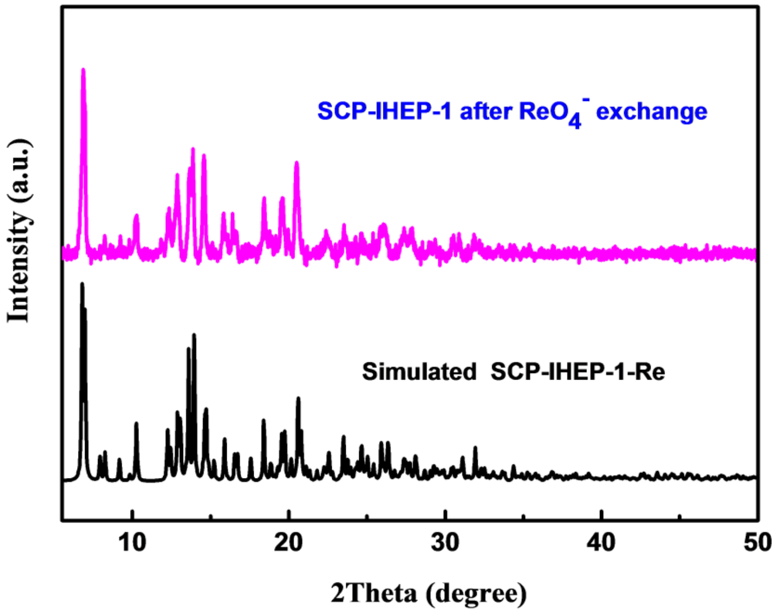

Supplementary Figure 19. A comparison of simulated PXRD pattern of crystalline SCP-IHEP-1-Re (black) and actual PXRD of SCP-IHEP-1 after ReO_4^- exchange (pink).

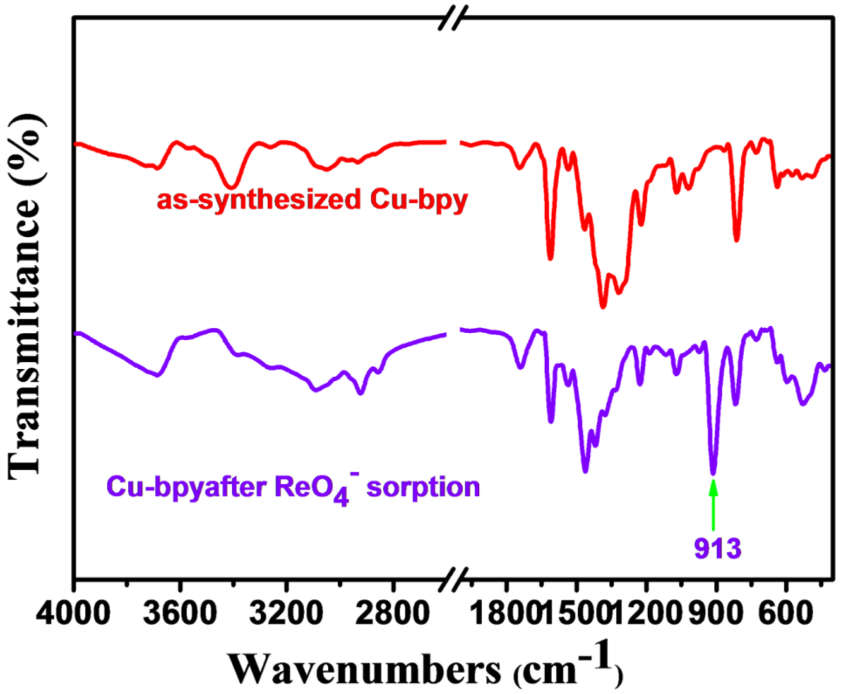

Supplementary Figure 20. IR spectra of Cu-bpy before (red) and after (purple) ReO_4^- exchange

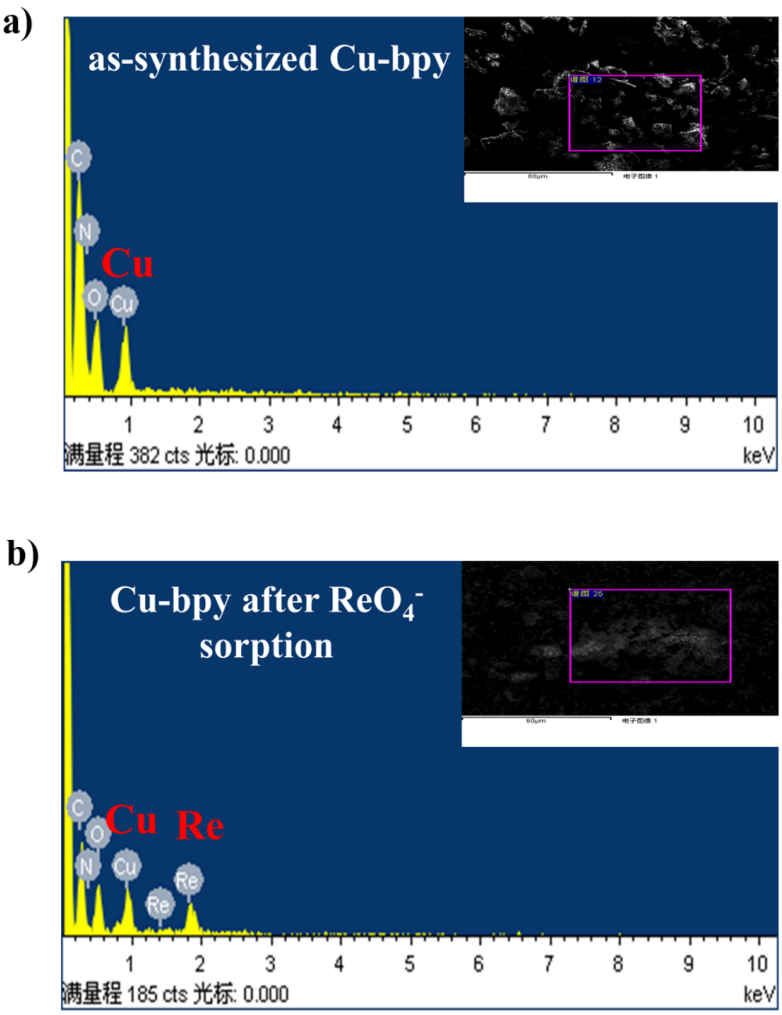

Supplementary Figure 21. EDS of Cu-bpy samples: (a) EDS before ReO_4^- exchange; (b) EDS after ReO_4^- exchange.

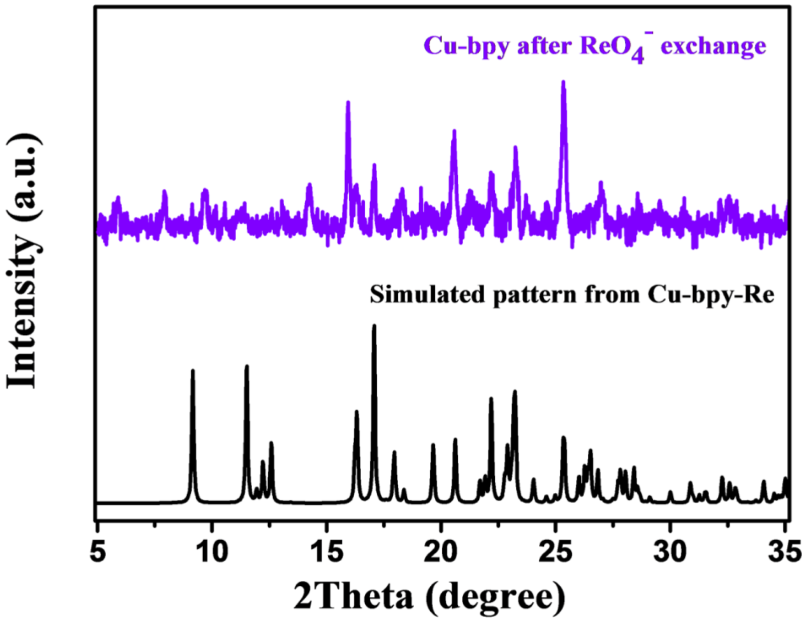

Supplementary Figure 22. A comparison of experimental PXRD of Cu-bpy after ReO_4^- exchange (purple) with simulated PXRD pattern of crystalline Cu-bpy-Re (black) suggesting a possible ReO_4^--induced transformation of Cu-bpy to Cu-bpy-Re.

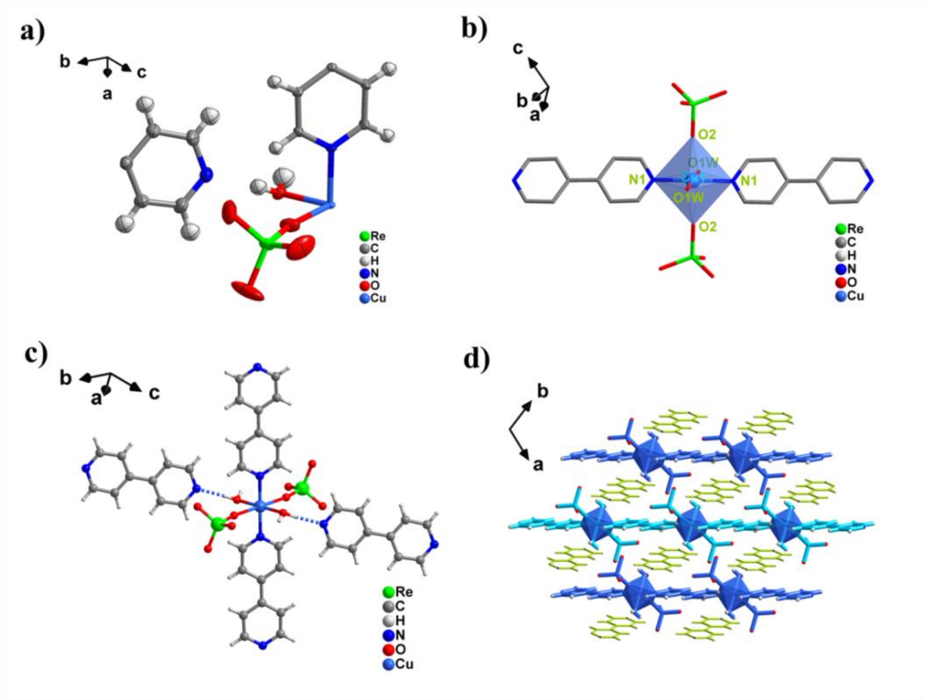

Supplementary Figure 23. Crystal structure of Cu-bpy-Re: a) ORTEP drawing of asymmetric unit with the 30% probability level for thermal ellipsoids; b) coordination sphere of six-coordinated Cu^{2+} node with deformed octahedral geometry; c) hydrogen bonding linkage between coordination sphere of Cu^{2+} and free 4, 4'-bipyridine moieties; d) 3D crystal stacking viewed from *c* axis.

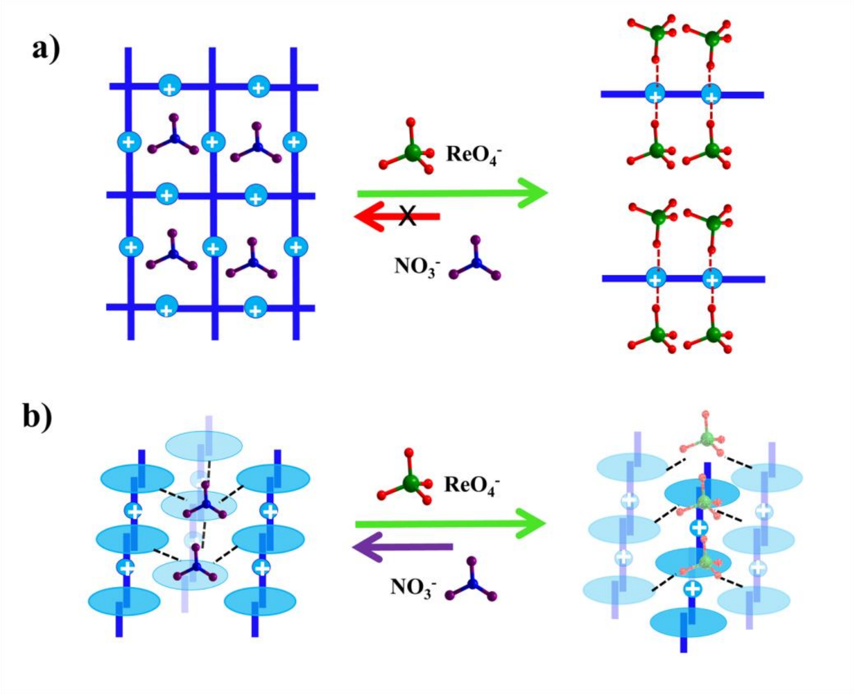

Supplementary Figure 24. A comparison of anion exchange mechanisms for two kinds of cationic materials: a) CB8-free MOM sorbents; b) the CB8-based MOM sorbent material in this work.

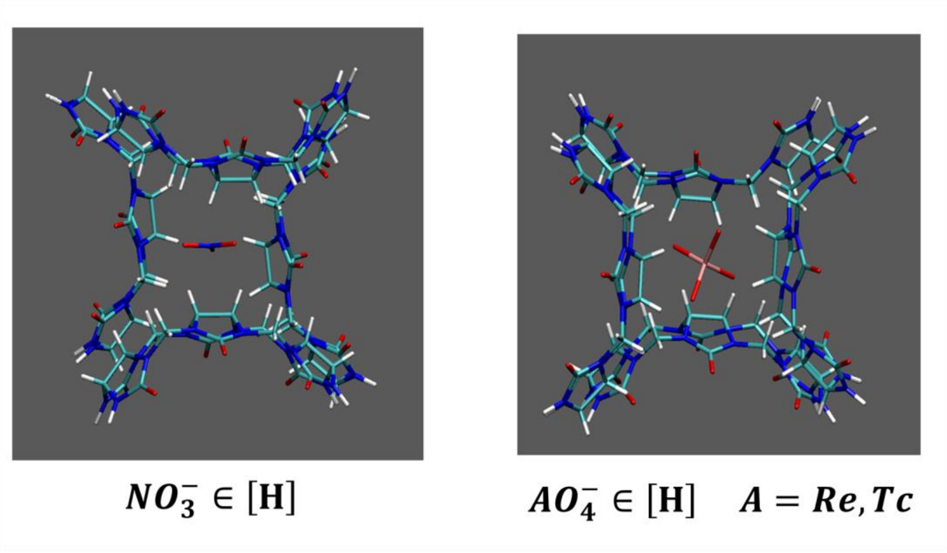

Supplementary Figure 25. Simplified models of the tetrahedral pores representing the key components [**H**] from four CB8 macrocycles for NO_3^- and ReO_4^-/TcO_4^- encapsulation: left, NO_3^- anion trapped in [**H**]; right, ReO_4^-/ TcO_4^- anion trapped in [**H**].

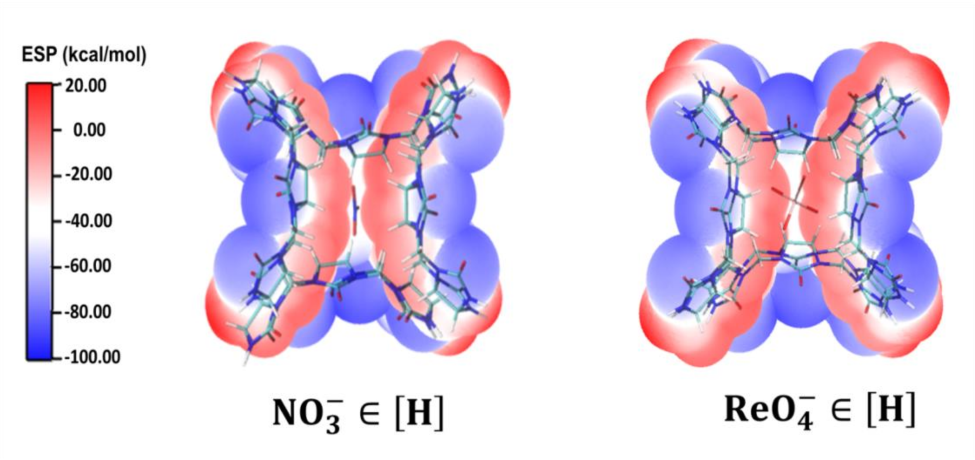

Supplementary Figure 26. ESP distributions for different models: (a) NO_3^- anion trapped in [**H**]; (b) ReO_4^- anion trapped in [**H**]. C, light blue; H, white; N, blue; O, red; Re, pink.

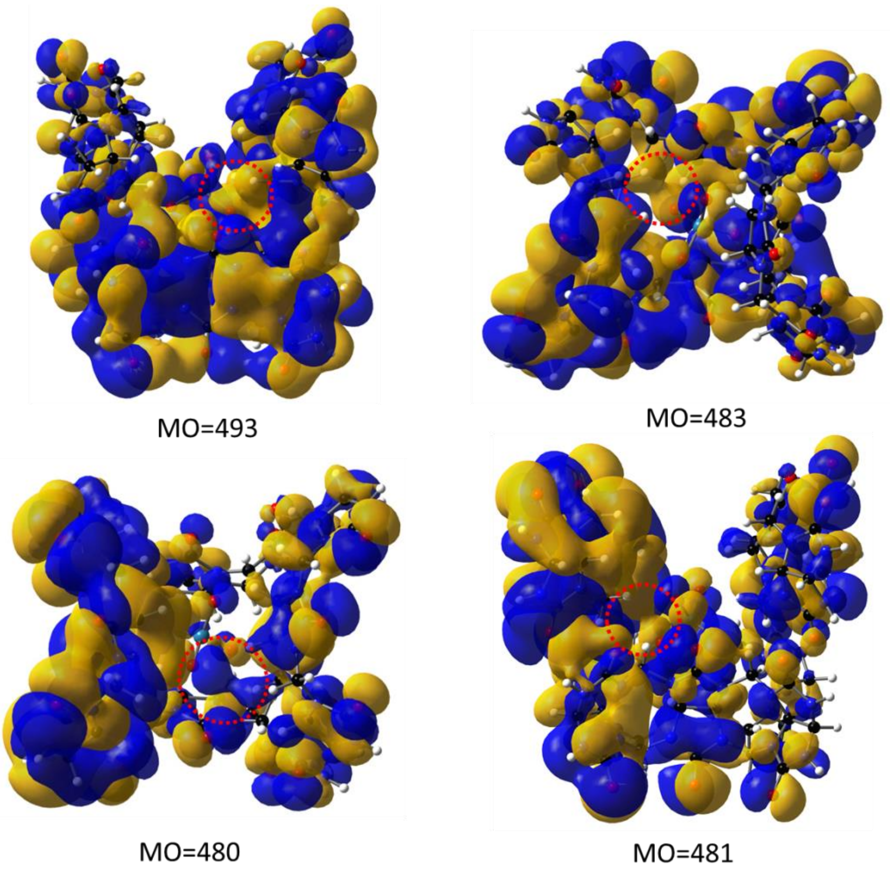

Supplementary Figure 27. Representative molecular orbitals contributing to hydrogen bonding between **[H]** and ReO_4^- (Red circles give the specific orbital interaction sites).

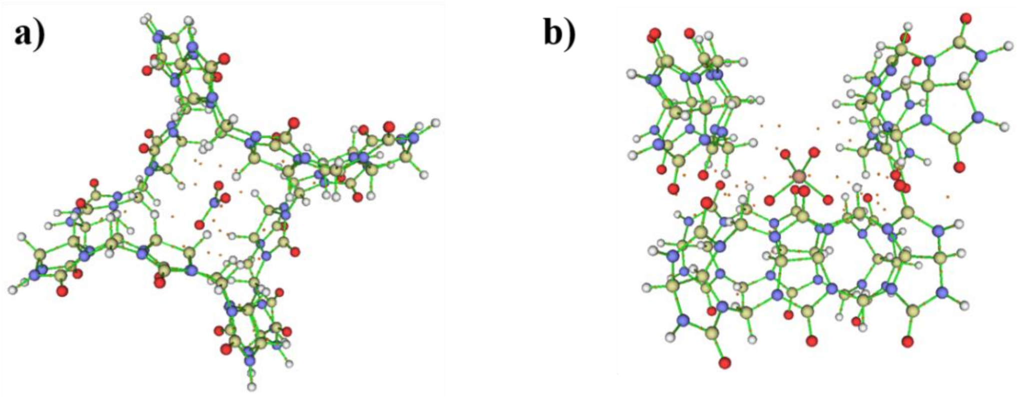

Supplementary Figure 28. Bond critical points (BCPs, small orange spheres) using QTAIM analysis.for different models: (a) CB8-based host **[H]** and NO_3^-; (b) CB8-based host **[H]** and ReO_4^-.

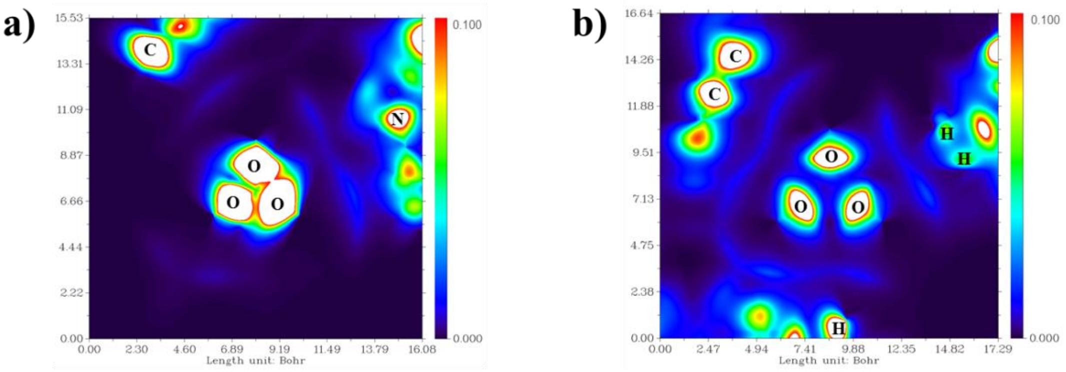

Supplementary Figure 29. The δ_g function (electron density gradient-based descriptor) maps using IGM analysis for different models: (a) CB8-based host **[H]** and NO_3^-; (b) CB8-based host **[H]** and ReO_4^-.

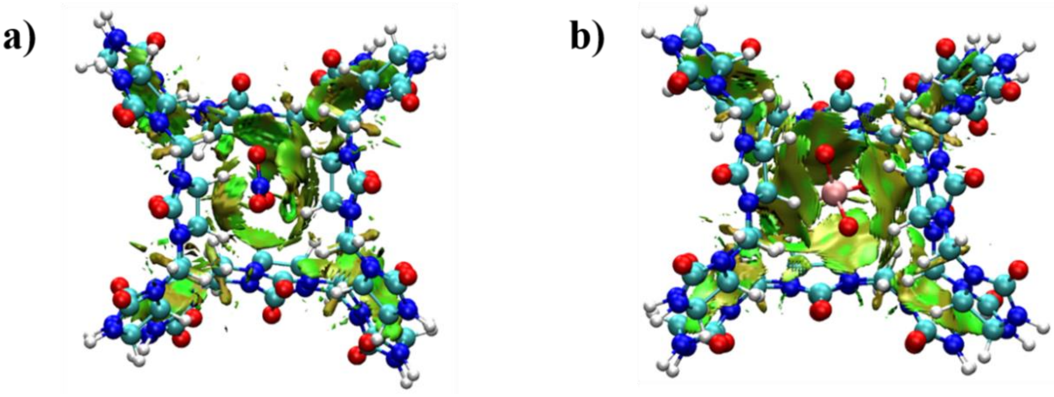

Supplementary Figure 30. Gradient isosurfaces (0.8 a.u.) based on the sign(λ_2)ρ values for different models using RDG analysis.: (a) CB8-based host **[H]** and NO_3^-; (b) CB8-based host **[H]** and ReO_4^-. Blue color represents a strong attraction, and red denotes a strong repulsion.

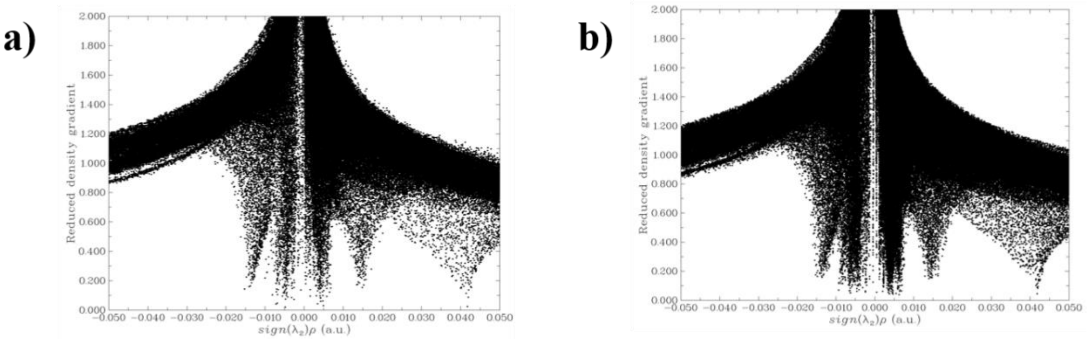

Supplementary Figure 31. The plots of RDG versus the electron density multiplied by the sign of the second Hessian eigenvalue (sign(λ_2)ρ) for different models: (a) CB8-based host **[H]** and NO_3^-; (b) CB8-based host **[H]** and ReO_4^-.

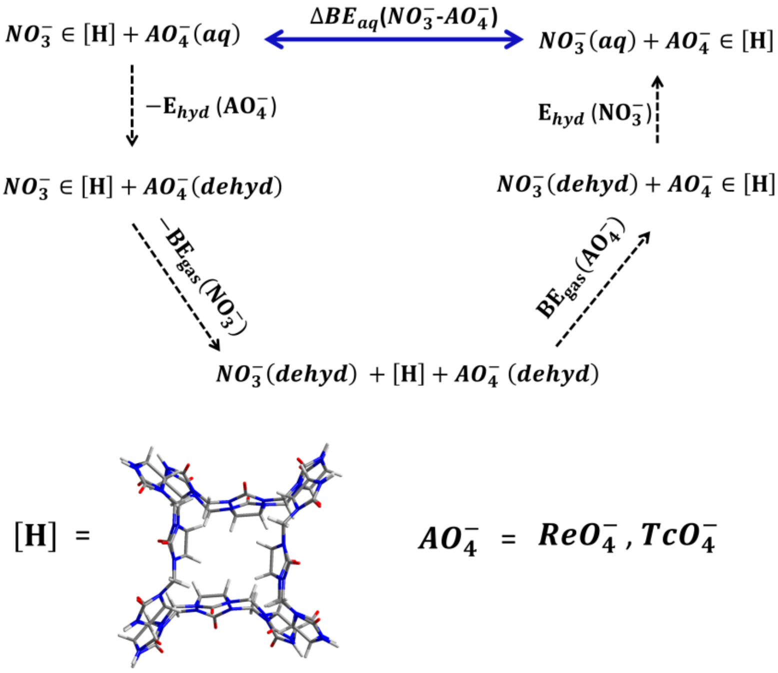

Supplementary Figure 32. Reaction paths for anion exchange of TcO_4^-/ReO_4^- by the host [H] as described by four steps: target TcO_4^- or ReO_4^- anion dehydration; dissociation of initial NO_3^- anion from [H]; encapsulation of target anion in [H]; and hydration of initial anion.

Supplementary Tables

Supplementary Table 1. Crystal data and structure refinement of all five compounds.

	2bpy@CB8	SCP-IHEP-1	SCP-IHEP-1-Re	Cu-bpy	Cu-bpy-Re
formula	$C_{68}H_{98}N_{36}O_{35}$	$C_{68}H_{88}N_{38}O_{44}Cu$	$C_{68}H_{64}N_{36}O_{36}CuRe_2$	$C_{40}H_{36}N_{10}O_9Cu_2$	$C_{20}H_{20}CuN_4O_{10}Re_2$
formula weight	1979.82	2205.30	2397.49	927.87	912.34
crystal system	monoclinic	monoclinic	monoclinic	orthorhombic	triclinic
space group	P 2_1/c	P 2_1/n	C 2/c	Fddd	P -1
a, Å	23.916(5)	18.3317(9)	26.188(5)	15.0740(14)	7.9898(4)
b, Å	16.498(3)	14.0853(7)	13.657(3)	41.699(4)	8.5301(5)
c, Å	22.729(5)	18.4417(9)	25.536(5)	41.878(4)	10.6048(6)
α, deg	90	90	90	90	105.065(2)
β, deg	106.31(3)	94.423(3)	96.36(3)	90	103.847(2)
γ, deg	90	90	90	90	106.060(2)
V, $Å^3$	8607(3)	4747.6(4)	9077(3)	26323(4)	631.83(6)
Z	4	2	4	16	1
T, K	153	170	153	293	297
F(000)	4152.0	2286.0	4764.0	7616.0	427.0
Dc, g cm^{-3}	1.528	1.543	1.754	0.937	2.398
μ (mm^{-1})	0.125	1.331	2.971	0.689	10.450
R_{int}	0.0551	0.0686	0.0737	0.0609	0.0386
R_1, wR_2 [I ≥ 2σ(I)]	0.0568, 0.1412	0.0887, 0.2438	0.1265, 0.3608	0.0710, 0.2126	0.0306, 0.0605
R_1, wR_2 (all data)	0.1107, 0.1690	0.1093, 0.2595	0.1474, 0.3737	0.0970, 0.2342	0.0400, 0.0654

Supplementary Table 2. Change of energies for reactions between bpy and CB8.

Reactions in aqueous solution	Δ*G*[a] (kcal mol^{-1})	Δ*H*[b] (kcal mol^{-1})	Δ*S*[c] (cal mol^{-1})
2bpy→ $(bpy)_2$	1.14	-9.62	-36.09
2bpy+ CB8→$(bpy)_2$@CB8 ($(bpy)_2$@CB8 model based on 2bpy@CB8 crystal structure)	-13.96	-44.98	-104.05
2bpy+ CB8→$(bpy)_2$@CB8 ($(bpy)_2$@CB8 model based on 2bpy@CB8 crystal structure, N(bpy)-fixed structure optimization)	-23.08	-57.74	-116.25
2bpy+ CB8→$(bpy)_2$@CB8 ($(bpy)_2$@CB8 model based on SCP-IHEP-1, N(bpy)-fixed structure optimization)	-25.48	-62.36	-123.68
2bpy+ CB8→$(bpy)_2$@CB8 ($(bpy)_2$@CB8 model based on SCP-IHEP-1-Re, N(bpy)-fixed structure optimization)	-29.30	-66.68	-125.38

[a] ΔG, change of Gibbs free energies;
[b] ΔH, change of enthalpies;
[c] ΔS: change of entropies.

Supplementary Table 3. Distances for hydrogen bonds between CB8 and bpy.

Motifs	Hydrogen bond (D-H ···A)	$d_{H\cdots A}$ (Å)	Motifs	Hydrogen bond (D-H ···A)	$d_{H\cdots A}$ (Å)
2bpy@CB8 (bpy-N1, N2)	C4-H4 ·O4	2.656(2)	2bpy@CB8 (bpy-N3, N4)	C11-H11 ·O14	2.911(2)
	C7-H7 ·O7	2.929(1)		C12-H12 ·O10	3.075(1)
	C10-H10 ·O5	2.613(2)		C17-H17 ·O11	2.628(2)
	C10-H10 ·O4	2.725(1)		C18-H18 ·O11	2.944(1)
				C15-H15 ·O12	3.138(2)
				C14-H14 ·O16	3.129 (2)
				C20-H20 ·O16	2.678 (2)
SCP-IHEP-1	C8-H8 ·O3	2.787 (5)	SCP-IHEP-1-Re	C8-H8 ·O5	2.678(15)
	C8-H8 ·O4	3.160 (4)		C8-H8 ·O4	2.991(14)
	C2-H2 ·O7	2.702 (4)		C2-H2 ·O1	2.836(13)
	C2-H2 ·O8	2.947 (4)		C1-H1 ·O2	3.061(13)
	C10-H10 ·O6	2.783 (3)		C9-H9 ·O2	2.960(13)
	C10-H10 ·O5	2.963 (4)		C9-H9 ·O3	3.075(14)
	C4-H4 ·O2	2.701 (3)		C4-H4 ·O6	2.806(17)

Supplementary Table 4. Anion-related hydrogen bonds in SCP-IHEP-1 and SCP-IHEP-1-Re.

Compound	Hydrogen bond (D-H ···A)	$d_{H\cdots A}$ (Å)
SCP-IHEP-1	C21-H21B…O11	3.079(14)
	C25-H25…O10	2.672(8)
	C25’-H25’…O10	2.414(8)
	C22-H22A…O10	3.074(8)
	C24-H24…O10	2.941(7)
	C22-H22A…O9	3.038(12)
	C24-H24…O9	3.117(10)
SCP-IHEP-1-Re	C34-H34B…O9	3.102(24)
	C34’-H34B’…O9	3.102(24)
	C32-H32…O9	2.939(26)
	C32’-H32’…O9	2.939(26)
	C16-H16B…O9	2.872(24)
	C16’-H16B’…O9	2.872(24)
	C20-H20…O10	2.692(14)
	C20’-H20’…O10	2.692(14)
	C22-H22B…O10	2.854(15)
	C28-H28B…O10	2.665(15)
	C28’-H28B’…O10	2.665(15)
	C19-H19…O10	3.145(13)
	C16-H16A…O10	3.054 (12)
	C16’-H16A’…O10	3.054 (12)

Supplementary Table 5. Derived kinetics model constants for ReO_4^- removal by SCP-IHEP-1.

Pseudo-first-order			Pseudo-second-order			Saturation capacity from experiment (mg g^{-1})
q_e(mg g^{-1})	k_1 (min^{-1})	R^2	q_e(mg g^{-1})	k_2 (g mg^{-1} min^{-1})	R^2	
5.48	0.0191	0.875	88.4	0.0179	0.999	88.4

Supplementary Table 6. Derived kinetics model constants for ReO_4^- removal by Cu-bpy.

Pseudo-first-order			Pseudo-second-order			Saturation capacity from experiment (mg g^{-1})
q_e(mg g^{-1})	k_1 (min^{-1})	R^2	q_e(mg g^{-1})	k_2 (g mg^{-1} min^{-1})	R^2	
9.50	0.0741	0.772	16.7	0.0425	0.996	17.0

Supplementary Table 7. Fitting results of the sorption isotherm curve of SCP-IHEP-1.

Langmuir			Freundlich		
q_m(mg g^{-1})*	k_L(L mg^{-1})	R^2	k_F(L$^{1/n}$ mg$^{1-1/n}$ g^{-1})	n	R^2
157	0.48	0.999	75.31	5.87	0.777

*q_m: Re maximum sorption capacity (formula weight: 186 g mol^{-1}), corresponding to ReO_4^- (formula weight: 250 g mol^{-1}) sorption capacity of 1.344*q_m (Re maximum sorption capacity is 157 mg g^{-1} corresponds to ReO_4^- sorption capacity of 211 mg g^{-1}).

Supplementary Table 8. The distribution coefficients (K_d) of SCP-IHEP-1 and Cu-bpy.

Material	Initial Concentration of Re (mg L^{-1})	Final Concentration of Re (mg L^{-1})	Final removal percentage (%)	K_d (mL g^{-1})
SCP-IHEP-1	21.8	0.416	98.1	$1.0*10^5$
	42	0.320	99.2	$2.6*10^5$
Cu-bpy	19.8	11.8	40.4	$1.4*10^3$
	38.3	26.1	31.9	$0.9*10^3$

Supplementary Table 9. Fitting results of the sorption isotherm curve of Cu-bpy.

Langmuir			Freundlich		
q_m(mg g^{-1})*	k_L(L mg^{-1})	R^2	k_F($L^{1/n}$ $mg^{1-1/n}$ g^{-1})	n	R^2
103	0.0143	0.964	5.45	1.95	0.938

* q_m: Re maximum sorption capacity (formula weight: 186 g mol^{-1}), corresponding to ReO_4^- (formula weight: 250 g mol^{-1}) sorption capacity of 1.344*q_m (Re maximum sorption capacity is 103 mg g^{-1} corresponds to ReO_4^- sorption capacity of 138 mg g^{-1}).

Supplementary Table 10. The reaction energies for ReO_4^- exchange by SCP-IHEP-1.

ΔH (kJ mol^{-1})	ΔS (J mol^{-1})	ΔG (kJ mol^{-1})				
		300 (K)	305 (K)	310 (K)	315 (K)	320 (K)
-25.76	-15.04	-21.25	-21.17	-21.09	-21.02	-20.94

Supplementary Table 11. ReO_4^- removal capacity of SCP-IHEP-1 in the presence of equivalent amount of competing anions.

Solution	Initial Concentration of Re (c_0, mg L^{-1})	Final Concentration of Re (c_e, mg L^{-1})	Final removal percentage (%)	K_d (mL g^{-1})
ReO_4^-	45.0	0.574	98.7	$1.5*10^5$
ReO_4^- + NO_3^-	45.2	0.722	98.4	$1.2*10^5$
ReO_4^- + SO_4^{2-}	44.9	0.656	98.5	$1.3*10^5$
ReO_4^- + CO_3^{2-}	44.5	0.819	98.2	$1.0*10^5$
ReO_4^- + PO_4^{3-}	44.1	0.538	98.8	$1.6*10^5$
ReO_4^- + ClO_4^-	44.3	3.803	91.4	$2.1*10^4$

Supplementary Table 12. Exchange experiments of ReO_4^- by SCP-IHEP-1 in the presence of varying amounts of NO_3^- as the competing ion.

The ratio of NO_3^-:ReO_4^-	Initial Concentration of Re (c_0, mg L^{-1})	Final Concentration of Re (c_e, mg L^{-1})	Final removal percentage (%)	K_d (mL g^{-1})
0:1	45.0	0.574	98.7	$1.5*10^5$
1:1	45.2	0.722	98.4	$1.3*10^5$
5:1	44.2	0.506	98.9	$1.7*10^5$
10:1	44.0	1.211	97.2	$7.1*10^4$
20:1	44.2	1.681	96.2	$5.1*10^4$
100:1	43.2	6.789	84.3	$1.1*10^4$

Supplementary Table 13. Exchange experiments of ReO_4^- by SCP-IHEP-1 in the presence of varying amounts of SO_4^{2-} as the competing ion.

The ratio of SO_4^{2-}:ReO_4^-	Initial Concentration of Re (c_0, mg L^{-1})	Final Concentration of Re (c_e, mg L^{-1})	Final removal percentage (%)	K_d (mL g^{-1})
0:1	45.0	0.574	98.7	$1.5*10^5$
1:1	44.1	0.851	98.1	$1.0*10^5$
10:1	44.0	1.014	97.7	$8.5*10^4$
100:1	43.3	1.314	97.0	$6.4*10^4$
1000:1	41.1	2.155	94.8	$3.6*10^4$
4000:1	41.0	3.454	91.6	$2.2*10^4$

Supplementary Table 14. Analysis of oxygen atom charges of NO_3^-, ReO_4^- and TcO_4^- trapped in a tetrahedral pore.

Anion	z	r (nm)	-z/r (nm^{-1})		Method		
					Bader	NPA	Mulliken
NO_3^-	-1	0.179[30]	5.587	O	-0.522	-0.541	-0.354
ReO_4^-	-1	0.260[30]	3.846	O	-0.829	-0.731	-0.396
TcO_4^-	-1	0.250[31]	4.000	O	-0.775	-0.605	-0.372

Supplementary Table 15. Selected H-Bond (O···H) lengths (d, Å) and corresponding electron density (ρ, e Å^{-3}), laplacian ($\nabla^2\rho$, e Å^{-5}), kinetic (G), potential (V) energy density (kJ mol^{-1} Bohr^{-3}) at the bond critical points (BCPs) for the model fragments with **[H]** and oxygen atoms from NO_3^- or ReO_4^- anion.

	d	ρ	$\nabla^2\rho$	G	V
	2.458	0.067	0.769	18.3	-15.7
NO_3^-	2.882	0.022	0.335	6.9	-4.7
	2.901	0.019	0.243	5.3	-4.0
	2.511	0.060	0.754	17.4	-14.3
ReO_4^-	2.716	0.032	0.366	8.5	-7.1
	3.055	0.016	0.217	4.5	-3.1

Supplementary Table 16. Calculated energies for the simplified model of a guest anion (NO_3^-, ReO_4^- and TcO_4^-) trapped in the tetrahedral pore.

	$^{a}BE_{aq}$ (kcal mol^{-1})	BE_{gas} (kcal mol^{-1})	E_{hyd} (kcal mol^{-1})	$^{b}\Delta BE_{aq}$ (kcal mol^{-1})	$^{c}\Delta BE_{gas}$ (kcal mol^{-1})	$^{d}\Delta E_{hyd}$ (kcal mol^{-1})
NO_3^-	-7.04	-74.36	-67.32	0	0	0
ReO_4^-	-17.82	-73.45	-55.63	-10.78	-0.91	-11.69
TcO_4^-	-17.48	-72.24	-54.76	-10.44	-2.12	-12.56

[a] $BE_{aq} = BE_{gas} - E_{hyd}$

[b] $\Delta BE_{aq} = BE_{gas}(AO_4^-) - BE_{gas}(NO_3^-) - E_{hyd}(AO_4^-) + E_{hyd}(NO_3^-) = BE_{aq}(AO_4^-) - BE_{aq}(NO_3^-)$

[c] $\Delta BE_{gas} = BE_{gas}(AO_4^-) - BE_{gas}(NO_3^-)$

[d] $\Delta E_{hyd} = -(E_{hyd} - E_{hyd}(NO_3^-))$

Supplementary References

1. Becke AD. Density-functional exchange-energy approximation with correct asymptotic behavior. *Phys. Rev. A* **38**, 3098 (1988).
2. Frisch MJ, *et al.* Gaussian 16 (ed^(eds). Revision B.01 edn. Gaussian, Inc. (2016).
3. Lan J-H, Shi W-Q, Yuan L-Y, Zhao Y-L, Li J, Chai Z-F. Trivalent Actinide and Lanthanide Separations by Tetradentate Nitrogen Ligands: A Quantum Chemistry Study. *Inorg. Chem.* **50**, 9230-9237 (2011).
4. Wang C-Z, Shi W-Q, Lan J-H, Zhao Y-L, Wei Y-Z, Chai Z-F. Complexation Behavior of Eu(III) and Am(III) with CMPO and Ph2CMPO Ligands: Insights from Density Functional Theory. *Inorg. Chem.* **52**, 10904-10911 (2013).
5. Wu Q-Y, *et al.* Understanding the Bonding Nature of Uranyl Ion and Functionalized Graphene: A Theoretical Study. *J. Phys. Chem. A* **118**, 2149-2158 (2014).
6. Wu Q-Y, *et al.* Theoretical Investigation on Multiple Bonds in Terminal Actinide Nitride Complexes. *Inorg. Chem.* **53**, 9607-9614 (2014).
7. Wu Q-Y, Lan J-H, Wang C-Z, Zhao Y-L, Chai Z-F, Shi W-Q. Terminal U≡E (E = N, P, As, Sb, and Bi) Bonds in Uranium Complexes: A Theoretical Perspective. *J. Phys. Chem. A* **119**, 922-930 (2015).
8. Perdew JP, Burke K, Ernzerhof M. Generalized Gradient Approximation Made Simple. *Phys. Rev. Lett.* **77**, 3865-3868 (1996).
9. Kresse G, Furthmuller J. Efficient iterative schemes for ab initio total-energy calculations using a plane-wave basis set. *J. Phys. Chem. B* **54**, 11169-11186 (1996).
10. Andrae D, Häußermann U, Dolg M, Stoll H, Preuß H. Energy-adjustedab initio pseudopotentials for the second and third row transition elements. *Theoret. Chim. Acta.* **77**, 123-141 (1990).
11. Martin JML, Sundermann A. Correlation consistent valence basis sets for use with the Stuttgart–Dresden–Bonn relativistic effective core potentials: The atoms Ga–Kr and In–Xe. *J. Chem. Phys.* **114**, 3408-3420 (2001).
12. Lu T, Chen F. Multiwfn: A multifunctional wavefunction analyzer. *J. Comput. Chem.* **33**, 580-592 (2012).
13. Bader RFW. *Atoms in Molecules-a Quantum Theory*. Oxford Univ. Press (1990).
14. Bader RFW. A Quantum-Theory of Molecular-Structure and Its Applications. *Chem. Rev.* **91**, 893-928 (1991).
15. Bader RFW. A bond path: A universal indicator of bonded interactions. *J. Phys. Chem. A* **102**, 7314-7323 (1998).
16. Bader RFW. Principle Of Stationary Action And the Definition Of a Proper Open System. *Phys. Rev. B* **49**, 13348-13356 (1994).
17. Becke AD. Density-Functional Thermochemistry .3. The Role Of Exact Exchange. *J. Chem. Phys.* **98**, 5648-5652 (1993).
18. Lee CT, Yang WT, Parr RG. Development Of the Colle-Salvetti Correlation-Energy Formula into a Functional Of the Electron-Density. *Phys. Rev. B* **37**, 785-789 (1988).
19. Grimme S, Antony J, Ehrlich S, Krieg H. A consistent and accurate ab initio parametrization of density functional dispersion correction (DFT-D) for the 94 elements H-Pu. *J. Chem. Phys.* **132**, 154104 (2010).
20. Cao XY, Dolg M. Segmented contraction scheme for small-core actinide pseudopotential basis

sets. *J. Mol. Struc.-Theochem.* **673**, 203-209 (2004).

21. Kuchle W, Dolg M, Stoll H, Preuss H. Energy-Adjusted Pseudopotentials for the Actinides - Parameter Sets And Test Calculations for Thorium And Thorium Monoxide. *J. Chem. Phys.* **100**, 7535-7542 (1994).
22. Dolg M, Stoll H, Preuss H. Energy-Adjusted Abinitio Pseudopotentials for the Rare-Earth Elements. *J. Chem. Phys.* **90**, 1730-1734 (1989).
23. Lefebvre C, Rubez G, Khartabil H, Boisson JC, Contreras-Garcia J, Henon E. Accurately extracting the signature of intermolecular interactions present in the NCI plot of the reduced density gradient versus electron density. *Phys. Chem. Chem. Phys.* **19**, 17928-17936 (2017).
24. Johnson ER, Keinan S, Mori-Sanchez P, Contreras-Garcia J, Cohen AJ, Yang WT. Revealing Noncovalent Interactions. *J. Am. Chem. Soc.* **132**, 6498-6506 (2010).
25. Lu T, Chen FW. Multiwfn: A multifunctional wavefunction analyzer. *J. Comput. Chem.* **33**, 580-592 (2012).
26. Humphrey W, Dalke A, Schulten K. VMD: Visual molecular dynamics. *J. Mol. Graph. Model.* **14**, 33-38 (1996).
27. Barone V, Cossi M. Quantum Calculation of Molecular Energies and Energy Gradients in Solution by a Conductor Solvent Model. *J. Phys. Chem. A* **102**, 1995-2001 (1998).
28. M. C, N. R, G. S, V. B. Energies, Structures, and Electronic Properties of Molecules in Solution with the C-PCM Solvation Model. *J. Comput. Chem.* **24**, 669-681. (2003).
29. Klamt A, Schüürmann G. COSMO: a new approach to dielectric screening in solvents with explicit expressions for the screening energy and its gradient. *J. Chem. Soc. Perkin Trans.* **2**, 799-805 (1995).
30. Marcus Y. Thermodynamics Of Solvation Of Ions .5. Gibbs Free-Energy Of Hydration at 298.15-K. *J. Chem. Soc. Faraday Trans.* **87**, 2995-2999 (1991).
31. Custelcean R, Moyer BA. Anion separation with metal-organic frameworks. *Eur. J. Inorg. Chem.*, 1321-1340 (2007).
